# Supplementary material for: Elevational shifts in reproductive ecology indicate the climate response of a model chasmophyte, Rainer’s bellflower (Campanula raineri)
Source: Ann Bot. 2024 Sep 30;135(1-2):181–98. doi: 10.1093/aob/mcae164 (PMC11805931; doi:10.1093/aob/mcae164)
Supplement: mcae164_suppl_Supplementary_Data [file mcae164_suppl_supplementary_data.zip › aob-24018-s06.docx]

I have some minor final comments with the goal of improving the manuscript presentation. I recommend making the presentation of the linear regression results more consistent and present them all together and separately from the ANOVAs results for the among population comparisons. I would also strongly recommend breaking down the first three pages of the discussion into separate paragraphs to facilitate reading and comprehension. I think the manuscript impact would be increased if the authors followed these recommendations.

Line 373 Heading should not be indented

> We have modified accordingly.

Line 416 Heading should not be indented

> We have modified accordingly.

Line 444 replace 'that' with 'for which'

> We have modified accordingly.

Line 577 I am not clear how Fig S3 illustrates variation in R scores among populations.

> Thank you for noticing this, it is an error due to the previous rewording of the text. The figure is Fig. S1, and we have now corrected this.

Lines 574-582. I would recommend presenting only the results of the regression with elevation in this paragraph. The next paragraph could present the ANOVA results. It would be less confusing to the reader. These two tests examine different questions. Going back and forth between the two is confusing and the reader will want to clearly see which variables had statistically significant regression with elevation.

> The results are presented with variables in the same order as data collection (each variable with its own analysis), which follows this rationale: first FRR and CSR strategy, related to the individual growth and relative investment in reproducing structures, then all the traits specifically related to sexual reproduction (pollen and seed quality). The reader would presumably expect to find the results presented in a consistent order with respect to the Methods section. We believe that maintaining a consistent order in the presentation of Methods and Results is the best solution.

You need to be consistent with how you present the results of the regression analyses (at times you only present the slope and p value, other times you also present the adjusted R2 value). Typically, the regression equation (for linear regression) is presented, together with the F, and P value, and the R2 value can be useful too). You present some variables but not others in Figures and it is not clear why. One suggestion would be to make a Figure with separate panels for each of the variables that were statistically significant in the linear regression analyses. It is fine not to present the logistic regression figures.

> We added the requested statistics for linear regressions.

We added regression equations to Table 3 and Table S2 for the regressions applied to the insect dataset.

We added R2 adj, F-statistic and regression equations to the captions of Fig.3 and Fig. S4B,C (for Fig. S4B it was not possible to include an F-statistic because the function lmrob - from the R package robustbase - used for this regression did not produce this value).

Perhaps we did not understand what you meant with the second suggestion about making separate panels, but as for TSMF (Fig.3) and SSM and NSF (Fig. S4) (i.e., all linear regressions directly concerning *C. raineri*) these are all statistically significant regressions. As for the regressions applied to the insect database, where we have both significant and non-significant regressions we preferred to keep the regression lines together to allow an overview of trends. However, we added asterisks to Fig. 4 and Fig. S6 to indicate significant regressions. Also, legends for these figures have been modified.

Discussion
Your first paragraph is almost three pages long. I strongly recommend breaking it down into separate paragraphs to make it easier for the reader.

> The first paragraph has been split in smaller sections.
---

Although the revision to 24018 is considered in good shape, the Handling Editor reccomends a few changes that will improve the readability and impact of the paper. Please adjust and send in as soon as possible and the paper will be accepted in full.

We now also ask you to supply your figures in final high resolution format and ask you to carefully read the below requirements.
