# Supplementary material for: Elevational shifts in reproductive ecology indicate the climate response of a model chasmophyte, Rainer’s bellflower (Campanula raineri)
Source: Ann Bot. 2024 Sep 30;135(1-2):181–98. doi: 10.1093/aob/mcae164 (PMC11805931; doi:10.1093/aob/mcae164)

**Elevational shifts in reproductive ecology indicate the climate response of a model chasmophyte, Rainer’s bellflower (*Campanula raineri*)**

***Annals of Botany***

Sara Villa^1,2^, Giulia Magoga^3^, Matteo Montagna^3,4^, Simon Pierce^2^

Affiliation of the authors:

^1^ Institute for Sustainable Plant Protection, National Research Council, via Madonna del Piano 10, 50019, Sesto Fiorentino, Italy

^2^ Department of Agricultural and Environmental Sciences - Production, Landscape, Agroenergy (DiSAA), University of Milan, via G. Celoria 2, 20133, Milan, Italy

^3^ Department of Agricultural Sciences, University of Naples “Federico II”, via Università 100, 80055, Portici, Italy

^4^ BAT Center ‑ Interuniversity Center for Studies on Bioinspired Agro‑Environmental Technology, University of Napoli “Federico II”, via Università 100, 80055, Portici, Italy

Corresponding author: [simon.pierce@unimi.it](mailto:simon.pierce@unimi.it)


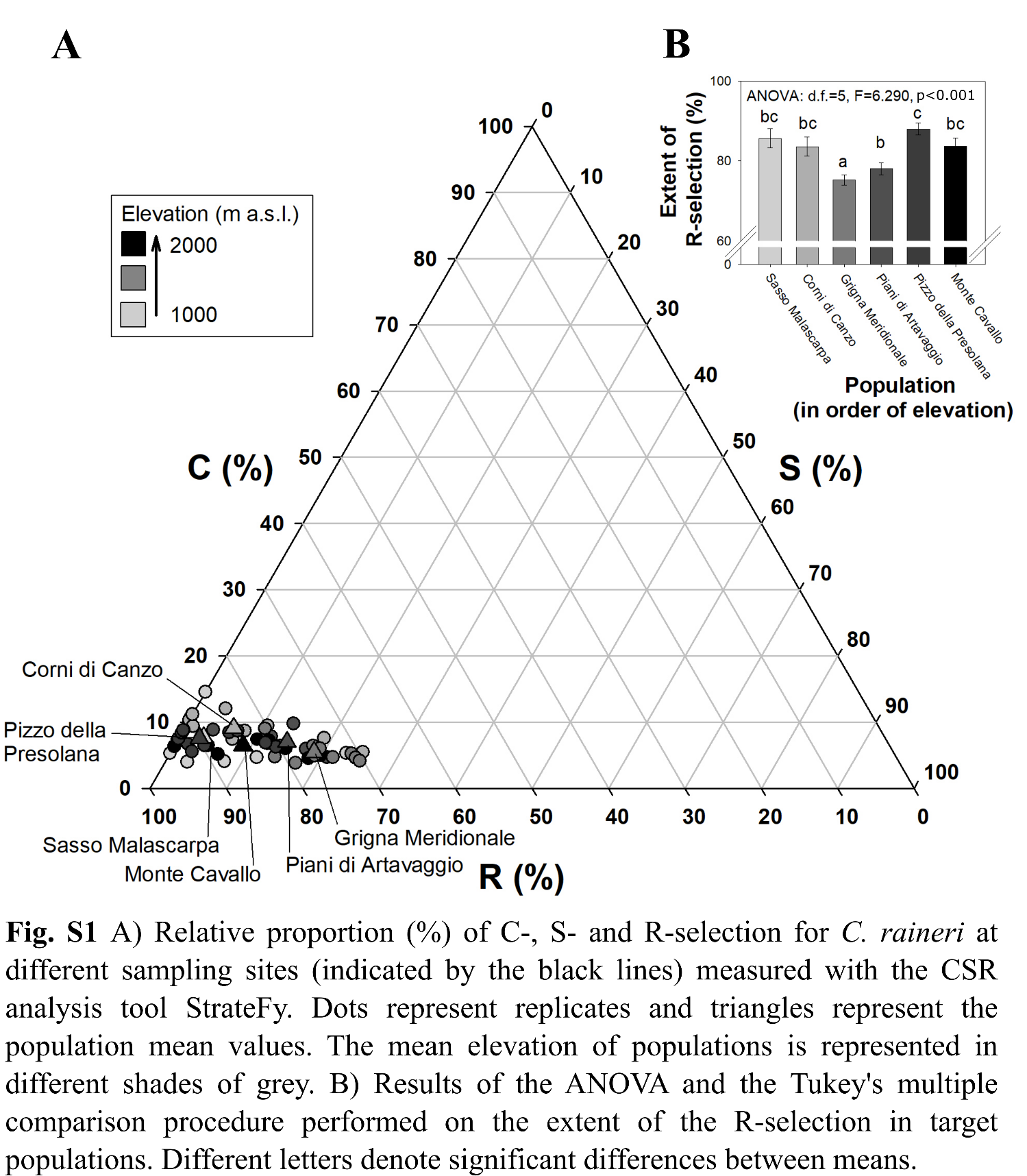


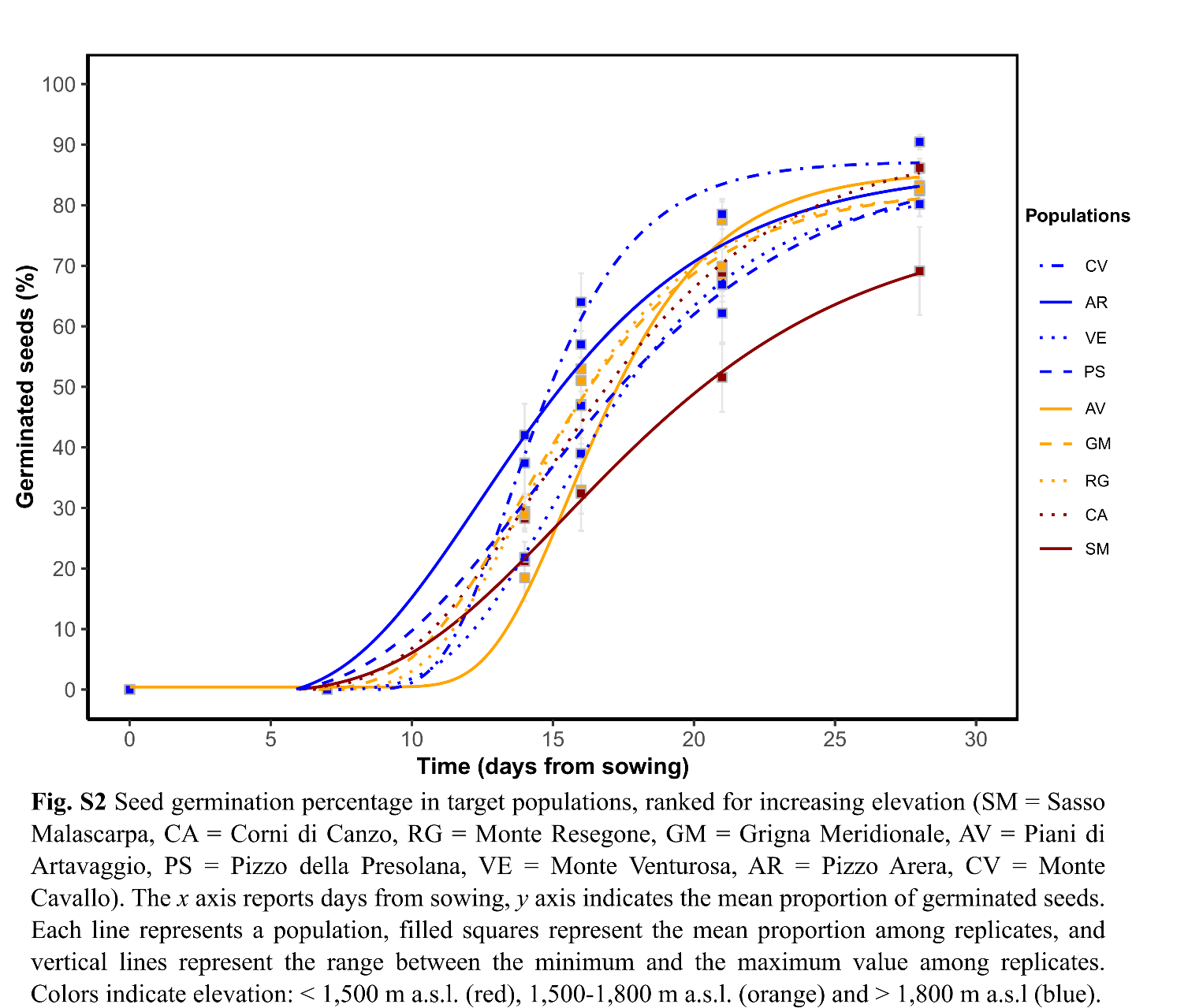


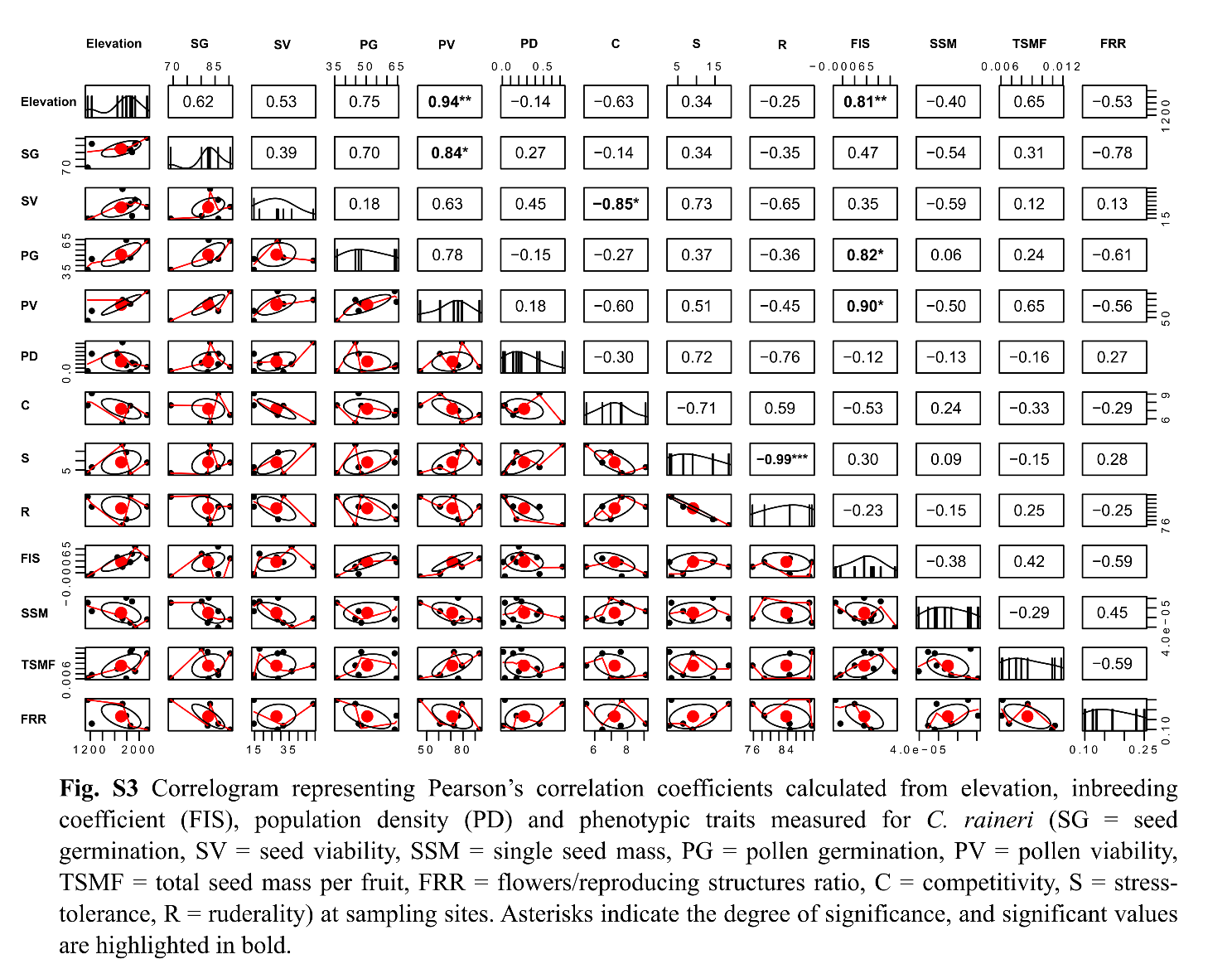


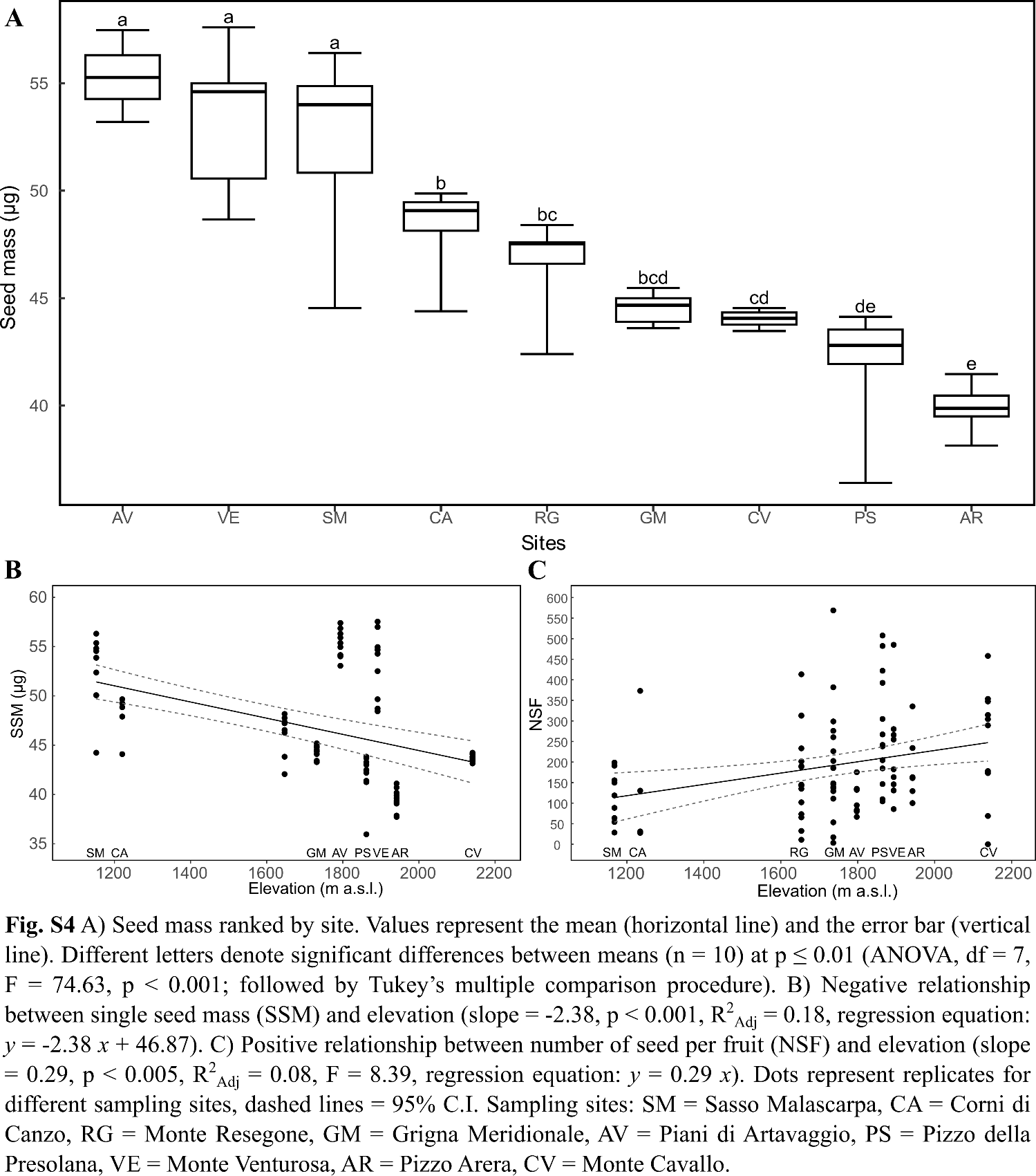


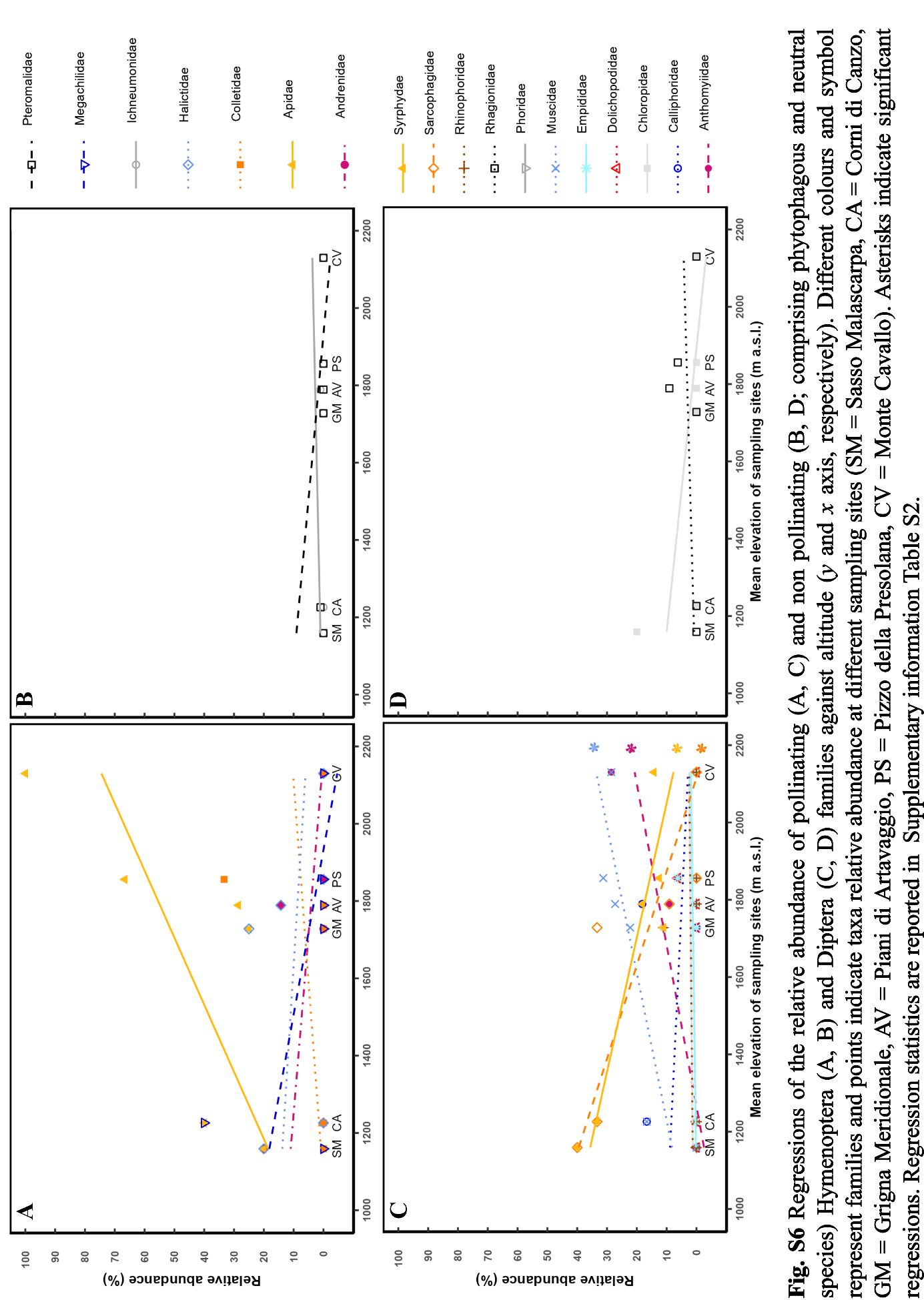

Supplement: mcae164_suppl_Supplementary_Data [file mcae164_suppl_supplementary_data.zip › aob-24018-s01.docx]
