# Supplementary material for: Elevational shifts in reproductive ecology indicate the climate response of a model chasmophyte, Rainer’s bellflower (Campanula raineri)
Source: Ann Bot. 2024 Sep 30;135(1-2):181–98. doi: 10.1093/aob/mcae164 (PMC11805931; doi:10.1093/aob/mcae164)
Supplement: mcae164_suppl_Supplementary_Data [file mcae164_suppl_supplementary_data.zip › aob-24018-s02.docx]

**Elevational shifts in reproductive ecology indicate the climate response of a model chasmophyte, Rainer’s bellflower (*Campanula raineri*)**

***Annals of Botany***

Sara Villa^1,2^, Giulia Magoga^3^, Matteo Montagna^3,4^, Simon Pierce^2^

Affiliation of the authors:

^1^ Institute for Sustainable Plant Protection, National Research Council, via Madonna del Piano 10, 50019, Sesto Fiorentino, Italy

^2^ Department of Agricultural and Environmental Sciences - Production, Landscape, Agroenergy (DiSAA), University of Milan, via G. Celoria 2, 20133, Milan, Italy

^3^ Department of Agricultural Sciences, University of Naples “Federico II”, via Università 100, 80055, Portici, Italy

^4^ BAT Center ‑ Interuniversity Center for Studies on Bioinspired Agro‑Environmental Technology, University of Napoli “Federico II”, via Università 100, 80055, Portici, Italy

Corresponding author: [simon.pierce@unimi.it](mailto:simon.pierce@unimi.it)

**Table S1** List of arthropods detected at the sampling sites (AV = Piani di Artavaggio, CA = Corni di Canzo, CV = Monte Cavallo, GM = Grigna Meridionale, PS = Pizzo della Presolana, SM = Sasso Malascarpa). For each record, sampling site and date (in the format d/m; year = 2021), taxonomic information (species, genus, family and order), identification method, ecology (only for taxa identified at least to genus level: 2 = pollinator, 1 = occasional pollinator, 0 = neutral (non-pollinator and non-phytophagous) species, -1 = phytophagous) and related bibliographical references are reported. For taxa identified with DNA barcoding the sequence ID in BoldSystems (https://www.boldsystems.org/) is reported. Taxa identified at least to genus level are highlighted in grey-filled cells.

| Locality | Date | Species name | Genus | Family | Order | Ecology | Method | BoldSystems record ID | Literature |
| --- | --- | --- | --- | --- | --- | --- | --- | --- | --- |
| AV | 23.07 | *Cleopomiarus graminis* Gyllenhal | *Cleopomiarus* | Curculionidae | Coleoptera | -1 | DNA barcoding | CRPOL042-23.COI-5P | Skuhrovec et al., 2018. ZooKeys 808:23-92;  Kajtoch et al., 2015. Mol. Ecol 24(15):4023-38 |
| AV | 23.07 | Curculionidae | nd | Curculionidae | Coleoptera | nd | photo |  |  |
| AV | 23.07 | *Dasytes* sp. | *Dasytes* | Melyridae | Coleoptera | -1 | DNA barcoding | CRPOL053-23.COI-5P | Weiss, Kaltenpoth, 2016. Front Microbiol 7:1486 |
| AV | 23.07 | Coleoptera | nd | nd | Coleoptera | nd | photo |  |  |
| AV | 23.07 | Curculionoidea | nd | nd | Coleoptera | nd | photo |  |  |
| AV | 23.07 | *Meligethes subrugosus* Gyllenhal | *Meligethes* | Nitidulidae | Coleoptera | -1 | DNA barcoding | CRPOL120-23.COI-5P | Audisio et al., 2014. Fragmenta entomologica 46(1-2):19-112 |
| AV | 23.07 | *Delia platura* Meigen | *Delia* | Anthomyiidae | Diptera | 2 | DNA barcoding | CRPOL044-23.COI-5P; CRPOL046-23.COI-5P | Wang, Chen, 2020. Plant Biology 23(1):111-20 |
| AV | 23.07 | *Calliphora* sp. | *Calliphora* | Calliphoridae | Diptera | 2 | photo |  | Clement et al., 2007. J Econ Entomol 100(1):131-5 |
| AV | 23.07 | *Calliphora vicina* Robineau-Desvoidy | *Calliphora* | [Calliphoridae](https://www.google.com/search?rlz=1C1DIMC_enIT834IT834&q=Calliphoridae&stick=H4sIAAAAAAAAAONgVuLUz9U3MEoztCx_xGjCLfDyxz1hKe1Ja05eY1Tl4grOyC93zSvJLKkUEudig7J4pbi5ELp4FrHyOifm5GQWZOQXZaYkpgIAFlpp9FQAAAA&sa=X&ved=2ahUKEwi56om7_db2AhXIS_EDHegSAOQQzIcDKAB6BAgaEAE) | Diptera | 2 | DNA barcoding | CRPOL038-23.COI-5P | Clement et al., 2007. J Econ Entomol 100(1):131-5 |
| AV | 23.07 | Calliphoridae sp.1 | nd | Calliphoridae | Diptera | nd | DNA barcoding | CRPOL050-23.COI-5P |  |
| AV | 23.07 | Dolichopodidae sp.1 | nd | Dolichopodidae | Diptera | nd | DNA barcoding | CRPOL051-23.COI-5P |  |
| AV | 23.07 | *Coenosia* sp. | *Coenosia* | Muscidae | Diptera | 1 | photo |  | Pont, 1995. Ber. nat.-med. Verein Innsbruck 82:311-18 |
| AV | 23.07 | Muscidae | nd | Muscidae | Diptera | nd | photo |  |  |
| AV | 23.07 | *Neomyia cornicina* Fabricius | *Neomyia* | Muscidae | Diptera | 2 | DNA barcoding | CRPOL039-23.COI-5P | Stone et al., 2017. Insect Pollinators Archive. DOI: 10.5519/0062900 |
| AV | 23.07 | *Phaonia* sp.2 | *Phaonia* | Muscidae | Diptera | 2 | DNA barcoding | CRPOL037-23.COI-5P | Biella et al., 2015. Studia dipterologica 22(1): 51–55 |
| AV | 23.07 | *Thricops nigritellus* Zetterstedt | *Thricops* | [Muscidae](https://www.google.com/search?rlz=1C1DIMC_enIT834IT834&q=Muscidae&stick=H4sIAAAAAAAAAONgVuLUz9U3sEzJsox_xGjCLfDyxz1hKe1Ja05eY1Tl4grOyC93zSvJLKkUEudig7J4pbi5ELp4FrFy-JYWJ2emJKYCAAjOG6NPAAAA&sa=X&ved=2ahUKEwjQvY-J_db2AhWJs6QKHVHTBggQzIcDKAB6BAgZEAE) | Diptera | 2 | DNA barcoding | CRPOL041-23.COI-5P; CRPOL043-23.COI-5P; CRPOL045-23.COI-5P; CRPOL047-23.COI-5P | Kühsel, 2015. Dissertation. https://tuprints.ulb.tu-darmstadt.de/5214/1/Dissertation%20Sara%20K%C3%BChsel_1.pdf |
| AV | 23.07 | Diptera | nd | nd | Diptera | nd | photo |  |  |
| AV | 23.07 | Diptera | nd | nd | Diptera | nd | photo |  |  |
| AV | 23.07 | *Rhagio* sp.1 | *Rhagio* | Rhagionidae | Diptera | 0 | DNA barcoding | CRPOL036-23.COI-5P | Lee, Suh, 2022. Journal of Asia-Pacific Biodiversity 15(3):370-74 |
| AV | 23.07 | Rhagionidae sp. | nd | Rhagionidae | Diptera | nd | DNA barcoding | CRPOL049-23.COI-5P |  |
| AV | 23.07 | *Sarcophaga okaliana* Lehrer | *Sarcophaga* | [Sarcophagidae](https://www.google.com/search?rlz=1C1DIMC_enIT834IT834&q=Sarcophagidae&stick=H4sIAAAAAAAAAONgVuLUz9U3MEozTMl6xGjCLfDyxz1hKe1Ja05eY1Tl4grOyC93zSvJLKkUEudig7J4pbi5ELp4FrHyBicWJecXZCSmZ6YkpgIASOM46VQAAAA&sa=X&ved=2ahUKEwiwxoPP_Nb2AhWMgP0HHRZvAcIQzIcDKAB6BAgaEAE) | Diptera | 2 | DNA barcoding | CRPOL040-23.COI-5P | Kühsel, 2015. Dissertation. https://tuprints.ulb.tu-darmstadt.de/5214/1/Dissertation%20Sara%20K%C3%BChsel_1.pdf |
| AV | 23.07 | *Eristalis tenax* L. | *Eristalis* | Syrphidae | Diptera | 2 | DNA barcoding, photo | CRPOL035-23.COI-5P | Jarlan et al., 1997. Journal Econ Entomol 90(6): 1650–54 |
| AV | 23.07 | Eristalini | nd | Syrphydae | Diptera | nd | photo |  |  |
| AV | 23.07 | *Eupeodes* sp. | *Eupeodes* | Syrphydae | Diptera | 2 | photo |  | Pekas et al., 2020. Biological Control 149: 104328 |
| AV | 23.07 | Syrphidae | nd | Syrphydae | Diptera | nd | photo |  |  |
| AV | 23.07 | *Andrena* sp. | *Andrena* | Andrenidae | Hymenoptera | 2 | photo |  | Tang et al., 2019. Biol Lett 15(10):20190479 |
| AV | 23.07 | *Apis mellifera* L. | *Apis* | Apidae | Hymenoptera | 2 | photo |  | Galimberti et al., 2014. PLoS One 9(10):e109363 |
| AV | 23.07 | *Bombus* sp.1 | *Bombus* | Apidae | Hymenoptera | 2 | photo |  | Wolf, Moritz, 2014. Apidologie 45(4):440-450 |
| AV | 23.07 | *Formica* sp. | *Formica* | Formicidae | Hymenoptera | 1 | photo |  | Rostás, Tautz 2010. In Seckbach & Dubinsky (eds), Springer. |
| AV | 23.07 | Formicidae | nd | Formicidae | Hymenoptera | nd | photo |  |  |
| AV | 23.07 | *Lasius emarginatus* Olivier | *Lasius* | Formicidae | Hymenoptera | 0 | photo |  | Marazzi et al., 2014. Bollettino della Società ticinese di scienze naturali 102:47-56 |
| AV | 23.07 | *Sphecodes geoffrellus* Kirby | *Sphecodes* | [Halictidae](https://www.google.com/search?rlz=1C1DIMC_enIT834IT834&biw=1536&bih=722&q=Halictidae&stick=H4sIAAAAAAAAAONgVuLUz9U3MLKwKC98xGjCLfDyxz1hKe1Ja05eY1Tl4grOyC93zSvJLKkUEudig7J4pbi5ELp4FrFyeSTmZCaXZKYkpgIAHubuD1EAAAA&sa=X&ved=2ahUKEwj40-qw_9b2AhWK7aQKHegTBE0QzIcDKAB6BAgeEAE) | Hymenoptera | 2 | DNA barcoding | CRPOL052-23.COI-5P | Wood et al., 2017. J Appl Ecol 54(1):323-33 |
| AV | 23.07 | *Ichneumon* sp. | *Ichneumon* | Ichneumonidae | Hymenoptera | 0 | DNA barcoding | CRPOL048-23.COI-5P | Tschopp et al., 2013. BMC Evolutionary Biology 13:74 |
| AV | 23.07 | *Erebia* sp. | *Erebia* | Nymphalidae | Lepidoptera | 2 | photo |  | Sistri et al., 2022. Insect Conserv &Diversity 15:136–48 |
| AV | 23.07 | *Thrips* sp. | *Thrips* | Thripidae | Thysanoptera | -1 | DNA barcoding | CRPOL118-23.COI-5P | Mound, Teulon, 1995. In Parker, Skinner & Lewis (eds). Springer;  Sperotto et al., 2019. Frontiers in Plant Science 10:866;  Carrillo-Arámbula et al., 2022. PLoS One 17(11): e0276865 |
| AV | 23.07 | *Balaustium* sp. | *Balaustium* | Erythraeidae | Trombidiformes | -1 | DNA barcoding | CRPOL119-23.COI-5P | Lowenberg, 1997. Oecologia 109(2):279-285 |
| AV | 23.07 | Trombidiformes | nd | nd | Trombidiformes | nd | photo |  |  |
| CA | 18.07 | *Cryptocephalus* sp. | *Cryptocephalus* | Chrysomelidae | Coleoptera | 1 | photo |  | Galloni, Cristofolini, 2003. Plant Syst & Evol 238(1/4):127-37 |
| CA | 18.07 | *Drilus flavescens* Olivier | *Drilus* | Elateridae | Coleoptera | 0 | DNA barcoding | CRPOL086-23.COI-5P | Bouchard, Bousquet, 2014. Ivy Press |
| CA | 18.07 | Coleoptera | nd | nd | Coleoptera | nd | photo |  |  |
| CA | 18.07 | *Pollenia pediculate* Macquart | *Pollenia* | [Calliphoridae](https://www.google.com/search?rlz=1C1DIMC_enIT834IT834&q=Calliphoridae&stick=H4sIAAAAAAAAAONgVuLUz9U3MEoztCx_xGjCLfDyxz1hKe1Ja05eY1Tl4grOyC93zSvJLKkUEudig7J4pbi5ELp4FrHyOifm5GQWZOQXZaYkpgIAFlpp9FQAAAA&sa=X&ved=2ahUKEwibytrZhtf2AhVMzaQKHVfvCWkQzIcDKAB6BAggEAE) | Diptera | 2 | DNA barcoding | CRPOL078-23.COI-5P; CRPOL079-23.COI-5P | Stone et al., 2017. Insect Pollinators Archive. DOI: 10.5519/0062900 |
| CA | 18.07 | Chloropidae | nd | Chloropidae | Diptera | nd | DNA barcoding | CRPOL076-23.COI-5P |  |
| CA | 18.07 | *Dolichopodidae* sp.2 | nd | Dolichopodidae | Diptera | nd | DNA barcoding | CRPOL080-23.COI-5P |  |
| CA | 18.07 | *Thricops* sp.2 | *Thricops* | [Muscidae](https://www.google.com/search?rlz=1C1DIMC_enIT834IT834&q=Muscidae&stick=H4sIAAAAAAAAAONgVuLUz9U3sEzJsox_xGjCLfDyxz1hKe1Ja05eY1Tl4grOyC93zSvJLKkUEudig7J4pbi5ELp4FrFy-JYWJ2emJKYCAAjOG6NPAAAA&sa=X&ved=2ahUKEwjQvY-J_db2AhWJs6QKHVHTBggQzIcDKAB6BAgZEAE) | Diptera | 2 | DNA barcoding | CRPOL085-23.COI-5P | Kühsel, 2015. Dissertation. https://tuprints.ulb.tu-darmstadt.de/5214/1/Dissertation%20Sara%20K%C3%BChsel_1.pdf |
| CA | 18.07 | *Sarcophaga* sp.2 | *Sarcophaga* | [Sarcophagidae](https://www.google.com/search?rlz=1C1DIMC_enIT834IT834&q=Sarcophagidae&stick=H4sIAAAAAAAAAONgVuLUz9U3MEozTMl6xGjCLfDyxz1hKe1Ja05eY1Tl4grOyC93zSvJLKkUEudig7J4pbi5ELp4FrHyBicWJecXZCSmZ6YkpgIASOM46VQAAAA&sa=X&ved=2ahUKEwiwxoPP_Nb2AhWMgP0HHRZvAcIQzIcDKAB6BAgaEAE) | Diptera | 2 | DNA barcoding | CRPOL087-23.COI-5P | Kühsel, 2015. Dissertation. https://tuprints.ulb.tu-darmstadt.de/5214/1/Dissertation%20Sara%20K%C3%BChsel_1.pdf |
| CA | 18.07 | *Sarcophaga* sp.4 | *Sarcophaga* | Sarcophagidae | Diptera | 2 | photo |  | Kühsel, 2015. Dissertation. https://tuprints.ulb.tu-darmstadt.de/5214/1/Dissertation%20Sara%20K%C3%BChsel_1.pdf |
| CA | 18.07 | *Merodon rufus* Meigen | *Merodon* | Syrphidae | Diptera | 2 | DNA barcoding | CRPOL077-23.COI-5P | Ricarte et al., 2017. PLoS ONE 12(12):e0189852 |
| CA | 18.07 | *Eupeodes* sp. | *Eupeodes* | Syrphydae | Diptera | 2 | photo |  | Pekas et al., 2020. Biological Control 149: 104328 |
| CA | 18.07 | Tachinidae sp.1 | nd | Tachinidae | Diptera | nd | DNA barcoding | CRPOL081-23.COI-5P; CRPOL083-23.COI-5P |  |
| CA | 18.07 | *Planaphrodes* sp. | *Planaphrodes* | Cicadellidae | Hemiptera | 0 | DNA barcoding | CRPOL075-23.COI-5P | Nast, 1987. Annales Zoologici Warszawa, 40:535-661 |
| CA | 18.07 | *Bombus* sp.1 | *Bombus* | Apidae | Hymenoptera | 2 | photo |  | Wolf, Moritz, 2014. Apidologie 45(4):440-450 |
| CA | 18.07 | *Xylocopa* sp. | *Xylocopa* | Apidae | Hymenoptera | 2 | photo |  | Sadeh et al., 2007. Apidologie 38(6):508-17 |
| CA | 18.07 | Formicidae | nd | Formicidae | Hymenoptera | nd | photo |  |  |
| CA | 18.07 | *Hoplitis mitis* Nylander | *Hoplitis* | [Megachilidae](https://www.google.com/search?rlz=1C1DIMC_enIT834IT834&q=Megachilidae&stick=H4sIAAAAAAAAAONgVuLUz9U3MKwsqsx-xGjCLfDyxz1hKe1Ja05eY1Tl4grOyC93zSvJLKkUEudig7J4pbi5ELp4FrHy-KamJyZnZOZkpiSmAgCiLN3lUwAAAA&sa=X&ved=2ahUKEwiomtXjhtf2AhVRyaQKHakxCaEQzIcDKAB6BAgaEAE) | Hymenoptera | 2 | DNA barcoding | CRPOL082-23.COI-5P | Brandt et al., 2017. Journal of Chemical Ecology 43:4–12 |
| CA | 18.07 | *Hoplitis* sp. | *Hoplitis* | Megachilidae | Hymenoptera | 2 | photo |  | Brandt et al., 2017. Journal of Chemical Ecology 43:4–12 |
| CA | 18.07 | *Pteromalus phycidis* Ashmead | *Pteromalus* | Pteromalidae | Hymenoptera | 0 | DNA barcoding | CRPOL084-23.COI-5P | Takagi, 1987. Oecologia 71(3):321-24 |
| CA | 18.07 | *Satyrium* sp. | *Satyrium* | Lycaenidae | Lepidoptera | 2 | photo |  | Johnson et al., 2011. Plant Systematics and Evolution 292(1):95-103 |
| CA | 18.07 | Thripidae | nd | Thripidae | Thysanoptera | nd | photo |  |  |
| CA | 18.07 | *Thrips flavus* Schrank | *Thrips* | Thripidae | Thysanoptera | -1 | DNA barcoding | CRPOL107-23.COI-5P | Mound, Teulon, 1995. In Parker, Skinner & Lewis (eds). Springer;  Sperotto et al., 2019. Frontiers in Plant Science 10:866;  Carrillo-Arámbula et al., 2022. PLoS One 17(11): e0276865 |
| CA | 18.07 | *Thrips* sp. | *Thrips* | Thripidae | Thysanoptera | -1 | DNA barcoding | CRPOL104-23.COI-5P; CRPOL105-23.COI-5P; CRPOL106-23.COI-5P | Mound, Teulon, 1995. In Parker, Skinner & Lewis (eds). Springer;  Sperotto et al., 2019. Frontiers in Plant Science 10:866;  Carrillo-Arámbula et al., 2022. PLoS One 17(11): e0276865 |
| CA | 18.07 | *Thrips tabaci* Lindeman | *Thrips* | Thripidae | Thysanoptera | -1 | DNA barcoding | CRPOL108-23.COI-5P; CRPOL109-23.COI-5P | Mound, Teulon, 1995. In Parker, Skinner & Lewis (eds). Springer;  Sperotto et al., 2019. Frontiers in Plant Science 10:866;  Carrillo-Arámbula et al., 2022. PLoS One 17(11): e0276865 |
| CA | 18.07 | *Balaustium* sp. | *Balaustium* | Erythraeidae | Trombidiformes | -1 | DNA barcoding | CRPOL102-23.COI-5P; CRPOL103-23.COI-5P | Lowenberg, 1997. Oecologia 109(2):279-285 |
| CA | 18.07 | Trombidiformes | nd | nd | Trombidiformes | nd | photo |  |  |
| CV | 29.07 | *Botanophila* sp. | *Botanophila* | Anthomyiidae | Diptera | 2 | DNA barcoding | CRPOL074-23.COI-5P | Nakonechnaya et al., 2021. Plant Ecol & Evol 154(1): 39-48 |
| CV | 29.07 | *Delia platura* Meigen | *Delia* | Anthomyiidae | Diptera | 2 | DNA barcoding | CRPOL069-23.COI-5P | Wang, Chen, 2020. Plant Biology 23(1):111-20 |
| CV | 29.07 | Diptera | nd | Diptera | Diptera | nd | photo |  |  |
| CV | 29.07 | Empidoidea | nd | Empidoidea | Diptera | nd | DNA barcoding | CRPOL121-23.COI-5P |  |
| CV | 29.07 | *Helina* sp.1 | *Helina* | Muscidae | Diptera | 2 | DNA barcoding | CRPOL067-23.COI-5P; CRPOL071-23.COI-5P | Stone et al., 2017. Insect Pollinators Archive. DOI: 10.5519/0062900 |
| CV | 29.07 | *Mydaea rufinervis* Pokorny | *Mydaea* | [Muscidae](https://www.google.com/search?rlz=1C1DIMC_enIT834IT834&biw=1536&bih=722&q=Muscidae&stick=H4sIAAAAAAAAAONgVuLUz9U3sEzJsox_xGjCLfDyxz1hKe1Ja05eY1Tl4grOyC93zSvJLKkUEudig7J4pbi5ELp4FrFy-JYWJ2emJKYCAAjOG6NPAAAA&sa=X&ved=2ahUKEwj62Kbl_tb2AhWQQvEDHYzPD3wQzIcDKAB6BAgfEAE) | Diptera | 1 | DNA barcoding | CRPOL068-23.COI-5P | Pont, 1995. Ber. nat.-med. Verein Innsbruck 82:311-18 |
| CV | 29.07 | *Thricops* sp.1 | *Thricops* | [Muscidae](https://www.google.com/search?rlz=1C1DIMC_enIT834IT834&q=Muscidae&stick=H4sIAAAAAAAAAONgVuLUz9U3sEzJsox_xGjCLfDyxz1hKe1Ja05eY1Tl4grOyC93zSvJLKkUEudig7J4pbi5ELp4FrFy-JYWJ2emJKYCAAjOG6NPAAAA&sa=X&ved=2ahUKEwjQvY-J_db2AhWJs6QKHVHTBggQzIcDKAB6BAgZEAE) | Diptera | 2 | DNA barcoding | CRPOL065-23.COI-5P; CRPOL070-23.COI-5P; CRPOL072-23.COI-5P; CRPOL073-23.COI-5P | Kühsel, 2015. Dissertation. https://tuprints.ulb.tu-darmstadt.de/5214/1/Dissertation%20Sara%20K%C3%BChsel_1.pdf |
| CV | 29.07 | Opomyzidae | nd | Opomyzidae | Diptera | nd | photo |  |  |
| CV | 29.07 | *Triphleba* sp. | *Triphleba* | Phoridae | Diptera | 1 | DNA barcoding | CRPOL066-23.COI-5P | Disney, 2012. Springer Science & Business Media |
| CV | 29.07 | *Eristalis tenax* L. | *Eristalis* | Syrphidae | Diptera | 2 | DNA barcoding | CRPOL064-23.COI-5P | Jarlan et al., 1997. Journal Econ Entomol 90(6): 1650–54 |
| CV | 29.07 | Syrphydae | nd | Syrphydae | Diptera | nd | photo |  |  |
| CV | 29.07 | *Bombus* sp.2 | *Bombus* | Apidae | Hymenoptera | 2 | photo |  | Wolf, Moritz, 2014. Apidologie 45(4):440-450 |
| CV | 29.07 | Megachilidae | nd | Megachilidae | Hymenoptera | nd | photo |  |  |
| CV | 29.07 | Bourletiellidae | nd | Bourletiellidae | Symphypleona | nd | DNA barcoding | CRPOL122-23.COI-5P |  |
| CV | 29.07 | Trombidiformes | nd | Erythraeidae | Trombidiformes | nd | photo |  |  |
| GM | 20.07 | Melyridae | nd | Melyridae | Coleoptera | nd | photo |  |  |
| GM | 20.07 | *Delia platura* Meigen | *Delia* | Anthomyiidae | Diptera | 2 | DNA barcoding | CRPOL094-23.COI-5P; CRPOL096-23.COI-5P; CRPOL100-23.COI-5P | Wang, Chen, 2020. Plant Biology 23(1):111-20 |
| GM | 20.07 | *Lasiambia palposa* Fallén | *Lasiambia* | Chloropidae | Diptera | 2 | DNA barcoding | CRPOL098-23.COI-5P | Weiner, 2016. Dissertation. https://tuprints.ulb.tu-darmstadt.de/5458/1/Dissertation_Christiane_Natalie_Weiner_zur_Abgabe_nach_der_Disputation_2016.pdf |
| GM | 20.07 | *Dolichopodidae* sp.1 | nd | Dolichopodidae | Diptera | nd | DNA barcoding | CRPOL097-23.COI-5P; CRPOL099-23.COI-5P |  |
| GM | 20.07 | *Helina* sp.2 | *Helina* | Muscidae | Diptera | 2 | DNA barcoding | CRPOL093-23.COI-5P | Stone et al., 2017. Insect Pollinators Archive. DOI: 10.5519/0062900 |
| GM | 20.07 | *Helina subvittata* Séguy | *Helina* | Muscidae | Diptera | 2 | DNA barcoding | CRPOL091-23.COI-5P | Stone et al., 2017. Insect Pollinators Archive. DOI: 10.5519/0062900 |
| GM | 20.07 | Diptera | nd | nd | Diptera | nd | photo |  |  |
| GM | 20.07 | *Rhinomorinia sarcophagina* Schiner | *Rhinomorinia* | Rhinophoridae | Diptera | 2 | DNA barcoding | CRPOL101-23.COI-5P | Kühsel, 2015. Dissertation. https://tuprints.ulb.tu-darmstadt.de/5214/1/Dissertation%20Sara%20K%C3%BChsel_1.pdf |
| GM | 20.07 | *Sarcophaga porrecta* Böttcher | *Sarcophaga* | [Sarcophagidae](https://www.google.com/search?rlz=1C1DIMC_enIT834IT834&q=Sarcophagidae&stick=H4sIAAAAAAAAAONgVuLUz9U3MEozTMl6xGjCLfDyxz1hKe1Ja05eY1Tl4grOyC93zSvJLKkUEudig7J4pbi5ELp4FrHyBicWJecXZCSmZ6YkpgIASOM46VQAAAA&sa=X&ved=2ahUKEwiwxoPP_Nb2AhWMgP0HHRZvAcIQzIcDKAB6BAgaEAE) | Diptera | 2 | DNA barcoding | CRPOL088-23.COI-5P | Kühsel, 2015. Dissertation. https://tuprints.ulb.tu-darmstadt.de/5214/1/Dissertation%20Sara%20K%C3%BChsel_1.pdf |
| GM | 20.07 | *Sarcophaga* sp.3 | *Sarcophaga* | [Sarcophagidae](https://www.google.com/search?rlz=1C1DIMC_enIT834IT834&q=Sarcophagidae&stick=H4sIAAAAAAAAAONgVuLUz9U3MEozTMl6xGjCLfDyxz1hKe1Ja05eY1Tl4grOyC93zSvJLKkUEudig7J4pbi5ELp4FrHyBicWJecXZCSmZ6YkpgIASOM46VQAAAA&sa=X&ved=2ahUKEwiwxoPP_Nb2AhWMgP0HHRZvAcIQzIcDKAB6BAgaEAE) | Diptera | 2 | DNA barcoding | CRPOL095-23.COI-5P | Kühsel, 2015. Dissertation. https://tuprints.ulb.tu-darmstadt.de/5214/1/Dissertation%20Sara%20K%C3%BChsel_1.pdf |
| GM | 20.07 | *Sarcophaga variegate* Scopoli | *Sarcophaga* | [Sarcophagidae](https://www.google.com/search?rlz=1C1DIMC_enIT834IT834&q=Sarcophagidae&stick=H4sIAAAAAAAAAONgVuLUz9U3MEozTMl6xGjCLfDyxz1hKe1Ja05eY1Tl4grOyC93zSvJLKkUEudig7J4pbi5ELp4FrHyBicWJecXZCSmZ6YkpgIASOM46VQAAAA&sa=X&ved=2ahUKEwiwxoPP_Nb2AhWMgP0HHRZvAcIQzIcDKAB6BAgaEAE) | Diptera | 2 | DNA barcoding | CRPOL092-23.COI-5P | Kühsel, 2015. Dissertation. https://tuprints.ulb.tu-darmstadt.de/5214/1/Dissertation%20Sara%20K%C3%BChsel_1.pdf |
| GM | 20.07 | Sciaridae | nd | Sciaridae | Diptera | nd | DNA barcoding | CRPOL111-23.COI-5P |  |
| GM | 20.07 | *Eupeodes* sp. | *Eupeodes* | Syrphydae | Diptera | 2 | photo |  | Pekas et al., 2020. Biological Control 149: 104328 |
| GM | 20.07 | *Apis mellifera* L. | *Apis* | Apidae | Hymenoptera | 2 | DNA barcoding | CRPOL089-23.COI-5P | Galimberti et al., 2014. PLoS One 9(10):e109363 |
| GM | 20.07 | *Formica* sp. | *Formica* | Formicidae | Hymenoptera | 1 | photo |  | Rostás, Tautz 2010. In Seckbach & Dubinsky (eds), Springer. |
| GM | 20.07 | Formicidae | nd | Formicidae | Hymenoptera | nd | photo |  |  |
| GM | 20.07 | *Temnothorax* sp. | *Temnothorax* | Formicidae | Hymenoptera | 1 | photo |  | Castro et al., 2013. Plant Ecology 214(10):1233-45 |
| GM | 20.07 | *Lasioglossum* sp. | *Lasioglossum* | [Halictidae](https://www.google.com/search?rlz=1C1DIMC_enIT834IT834&q=Halictidae&stick=H4sIAAAAAAAAAONgVuLUz9U3MLKwKC98xGjCLfDyxz1hKe1Ja05eY1Tl4grOyC93zSvJLKkUEudig7J4pbi5ELp4FrFyeSTmZCaXZKYkpgIAHubuD1EAAAA&sa=X&ved=2ahUKEwjz2qCq_db2AhWyQvEDHTolDw4QzIcDKAB6BAgqEAE) | Hymenoptera | 2 | DNA barcoding, photo | CRPOL090-23.COI-5P | Zaninotto, Dajoz, 2022. Animals 12:923 |
| GM | 20.07 | Sarcoptiformes | nd | nd | Sarcoptiformes | nd | DNA barcoding | CRPOL110-23.COI-5P |  |
| GM | 20.07 | Balaustium sp. | *Balaustium* | Erythraeidae | Trombidiformes | -1 | DNA barcoding | CRPOL112-23.COI-5P | Lowenberg, 1997. Oecologia 109(2):279-285 |
| GM | 20.07 | Trombidiformes | nd | nd | Trombidiformes | nd | photo |  |  |
| PS | 02.08 | Coleoptera | nd | nd | Coleoptera | nd | photo |  |  |
| PS | 02.08 | *Eusphalerum* sp. | *Eusphalerum* | Staphylinidae | Coleoptera | 0 | DNA barcoding | CRPOL029-23.COI-5P | Betz et al., 2018. Springer. |
| PS | 02.08 | *Delia platura* | *Delia* | Anthomyiidae | Diptera | 2 | DNA barcoding | CRPOL013-23.COI-5P | Wang, Chen, 2020. Plant Biology 23(1):111-20 |
| PS | 02.08 | *Hercostomus* sp.1 | *Hercostomus* | [Dolichopodidae](https://www.google.com/search?rlz=1C1DIMC_enIT834IT834&biw=1536&bih=722&q=Dolichopodidae&stick=H4sIAAAAAAAAAONgVuLUz9U3SMstysl-xGjCLfDyxz1hKe1Ja05eY1Tl4grOyC93zSvJLKkUEudig7J4pbi5ELp4FrHyueTnZCZn5Bfkp2SmJKYCAGzSR5FVAAAA&sa=X&ved=2ahUKEwih0OiF_9b2AhXDzKQKHRysC3IQzIcDKAB6BAgmEAE) | Diptera | 2 | DNA barcoding | CRPOL017-23.COI-5P; CRPOL032-23.COI-5P | Buchmann et al., 2010. Project report. https://www.researchgate.net/publication/235099093_A_Study_of_Insect_Pollinators_Associated_with_DoD_TER-S_Flowering_Plants_Including_Identification_of_Habitat_Types_Where_They_Co-Occur_by_Military_Installation_in_the_Western_United_States |
| PS | 02.08 | *Hilara* sp. | *Hilara* | [Empididae](https://www.google.com/search?rlz=1C1DIMC_enIT834IT834&biw=1536&bih=722&q=Empididae&stick=H4sIAAAAAAAAAONgVuLUz9U3SCvMyrJ8xGjCLfDyxz1hKe1Ja05eY1Tl4grOyC93zSvJLKkUEudig7J4pbi5ELp4FrFyuuYWZKZkpiSmAgBZO6zCUAAAAA&sa=X&ved=2ahUKEwjnlqDV_tb2AhVBSPEDHXD_DEwQzIcDKAB6BAgdEAE) | Diptera | 1 | DNA barcoding | CRPOL030-23.COI-5P | Willis, Burkill, 1903. Annals of Botany 17(2):313–50 |
| PS | 02.08 | *Rhamphomyia* sp. | *Rhamphomyia* | [Empididae](https://www.google.com/search?rlz=1C1DIMC_enIT834IT834&biw=1536&bih=722&q=Empididae&stick=H4sIAAAAAAAAAONgVuLUz9U3SCvMyrJ8xGjCLfDyxz1hKe1Ja05eY1Tl4grOyC93zSvJLKkUEudig7J4pbi5ELp4FrFyuuYWZKZkpiSmAgBZO6zCUAAAAA&sa=X&ved=2ahUKEwjnieG7_tb2AhV1SfEDHUR8DF8QzIcDKAB6BAgmEAE) | Diptera | 2 | DNA barcoding | CRPOL007-23.COI-5P | Kullenberg, 1950. Oikos 2(1):1-19 |
| PS | 02.08 | *Coenosia obscuricula* Rondani | *Coenosia* | Muscidae | Diptera | 1 | DNA barcoding | CRPOL022-23.COI-5P; CRPOL034-23.COI-5P | Pont, 1995. Ber. nat.-med. Verein Innsbruck 82:311-18 |
| PS | 02.08 | *Coenosia* sp. | *Coenosia* | [Muscidae](https://www.google.com/search?rlz=1C1DIMC_enIT834IT834&biw=1536&bih=722&q=Muscidae&stick=H4sIAAAAAAAAAONgVuLUz9U3sEzJsox_xGjCLfDyxz1hKe1Ja05eY1Tl4grOyC93zSvJLKkUEudig7J4pbi5ELp4FrFy-JYWJ2emJKYCAAjOG6NPAAAA&sa=X&ved=2ahUKEwicwsbD_tb2AhXyS_EDHSXjCqUQzIcDKAB6BAgXEAE) | Diptera | 1 | DNA barcoding | CRPOL009-23.COI-5P | Pont, 1995. Ber. nat.-med. Verein Innsbruck 82:311-18 |
| PS | 02.08 | *Drymeia hamata* Fallén | *Drymeia* | Muscidae | Diptera | 2 | DNA barcoding | CRPOL002-23.COI-5P; CRPOL019-23.COI-5P | Willis, Burkill, 1903. Annals of Botany 17(2):313–50 |
| PS | 02.08 | *Drymeia* sp. | *Drymeia* | Muscidae | Diptera | 1 | DNA barcoding | CRPOL001-23.COI-5P | Willis, Burkill, 1903. Annals of Botany 17(2):313–50 |
| PS | 02.08 | Muscidae | nd | Muscidae | Diptera | nd | photo |  |  |
| PS | 02.08 | *Mydaea rufinervis* Pokorny | *Mydaea* | [Muscidae](https://www.google.com/search?rlz=1C1DIMC_enIT834IT834&biw=1536&bih=722&q=Muscidae&stick=H4sIAAAAAAAAAONgVuLUz9U3sEzJsox_xGjCLfDyxz1hKe1Ja05eY1Tl4grOyC93zSvJLKkUEudig7J4pbi5ELp4FrFy-JYWJ2emJKYCAAjOG6NPAAAA&sa=X&ved=2ahUKEwj62Kbl_tb2AhWQQvEDHYzPD3wQzIcDKAB6BAgfEAE) | Diptera | 1 | DNA barcoding | CRPOL025-23.COI-5P | Pont, 1995. Ber. nat.-med. Verein Innsbruck 82:311-18 |
| PS | 02.08 | *Phaonia* sp.1 | *Phaonia* | Muscidae | Diptera | 2 | DNA barcoding | CRPOL005-23.COI-5P; CRPOL021-23.COI-5P | Biella et al., 2015. Studia dipterologica 22(1): 51–55 |
| PS | 02.08 | *Phaonia* sp.2 | *Phaonia* | Muscidae | Diptera | 2 | DNA barcoding | CRPOL010-23.COI-5P; CRPOL026-23.COI-5P | Biella et al., 2015. Studia dipterologica 22(1): 51–55 |
| PS | 02.08 | *Thricops nigritellus* Zetterstedt | *Thricops* | [Muscidae](https://www.google.com/search?rlz=1C1DIMC_enIT834IT834&q=Muscidae&stick=H4sIAAAAAAAAAONgVuLUz9U3sEzJsox_xGjCLfDyxz1hKe1Ja05eY1Tl4grOyC93zSvJLKkUEudig7J4pbi5ELp4FrFy-JYWJ2emJKYCAAjOG6NPAAAA&sa=X&ved=2ahUKEwjQvY-J_db2AhWJs6QKHVHTBggQzIcDKAB6BAgZEAE) | Diptera | 2 | DNA barcoding | CRPOL011-23.COI-5P; CRPOL027-23.COI-5P | Kühsel, 2015. Dissertation thesis. https://tuprints.ulb.tu-darmstadt.de/5214/1/Dissertation%20Sara%20K%C3%BChsel_1.pdf |
| PS | 02.08 | *Thricops* sp.1 | *Thricops* | [Muscidae](https://www.google.com/search?rlz=1C1DIMC_enIT834IT834&q=Muscidae&stick=H4sIAAAAAAAAAONgVuLUz9U3sEzJsox_xGjCLfDyxz1hKe1Ja05eY1Tl4grOyC93zSvJLKkUEudig7J4pbi5ELp4FrFy-JYWJ2emJKYCAAjOG6NPAAAA&sa=X&ved=2ahUKEwjQvY-J_db2AhWJs6QKHVHTBggQzIcDKAB6BAgZEAE) | Diptera | 2 | DNA barcoding | CRPOL015-23.COI-5P; CRPOL018-23.COI-5P; CRPOL023-23.COI-5P | Kühsel, 2015. Dissertation thesis. https://tuprints.ulb.tu-darmstadt.de/5214/1/Dissertation%20Sara%20K%C3%BChsel_1.pdf |
| PS | 02.08 | Diptera | nd | nd | Diptera | nd | photo |  |  |
| PS | 02.08 | Phoridae sp.1 | nd | Phoridae | Diptera | nd | DNA barcoding | CRPOL012-23.COI-5P; CRPOL033-23.COI-5P |  |
| PS | 02.08 | *Rhagio* sp.1 | *Rhagio* | Rhagionidae | Diptera | 0 | DNA barcoding | CRPOL003-23.COI-5P | Lee, Suh, 2022. Journal of Asia-Pacific Biodiversity 15(3):370-74 |
| PS | 02.08 | *Cheilosia* sp. | *Cheilosia* | Syrphidae | Diptera | 2 | DNA barcoding | CRPOL016-23.COI-5P | Lucas et al., 2018. Sci Rep 8:5133 |
| PS | 02.08 | *Eristalis tenax* L. | *Eristalis* | Syrphidae | Diptera | 2 | DNA barcoding | CRPOL004-23.COI-5P; CRPOL006-23.COI-5P; CRPOL020-23.COI-5P | Jarlan et al., 1997. Journal Econ Entomol 90(6): 1650–54 |
| PS | 02.08 | *Apis mellifera* L. | *Apis* | Apidae | Hymenoptera | 2 | DNA barcoding | CRPOL024-23.COI-5P | Galimberti et al., 2014. PLoS One 9(10):e109363 |
| PS | 02.08 | *Bombus* sp.3 | *Bombus* | Apidae | Hymenoptera | 2 | photo |  | Wolf, Moritz, 2014. Apidologie 45(4):440-450 |
| PS | 02.08 | *Hylaeus nivalis* Morawitz | *Hylaeus* | [Colletidae](https://www.google.com/search?rlz=1C1DIMC_enIT834IT834&biw=1536&bih=722&q=Colletidae&stick=H4sIAAAAAAAAAONgVuLUz9U3SDaMN6h6xGjCLfDyxz1hKe1Ja05eY1Tl4grOyC93zSvJLKkUEudig7J4pbi5ELp4FrFyOefn5KSWZKYkpgIAWBAJkFEAAAA&sa=X&ved=2ahUKEwjP2OPJ_tb2AhUYRfEDHbmpCdQQzIcDKAB6BAggEAE) | Hymenoptera | 2 | DNA barcoding | CRPOL014-23.COI-5P; CRPOL028-23.COI-5P; CRPOL031-23.COI-5P | Falk, 2017. DOI: 10.13140/RG.2.2.33103.20644 |
| PS | 02.08 | Pteromalidae sp. | nd | Pteromalidae | Hymenoptera | nd | DNA barcoding | CRPOL008-23.COI-5P |  |
| SM | 11.07 | *Dasyopa triangulate* Becker | *Dasyopa* | [Chloropidae](https://www.faunaitalia.it/checklist/invertebrates/families/Chloropidae.html) | Diptera | 0 | DNA barcoding | CRPOL063-23.COI-5P | Merritt et al., 2009. Elsevier Inc. |
| SM | 11.07 | *Sarcophaga* sp.1 | *Sarcophaga* | [Sarcophagidae](https://www.google.com/search?rlz=1C1DIMC_enIT834IT834&q=Sarcophagidae&stick=H4sIAAAAAAAAAONgVuLUz9U3MEozTMl6xGjCLfDyxz1hKe1Ja05eY1Tl4grOyC93zSvJLKkUEudig7J4pbi5ELp4FrHyBicWJecXZCSmZ6YkpgIASOM46VQAAAA&sa=X&ved=2ahUKEwiwxoPP_Nb2AhWMgP0HHRZvAcIQzIcDKAB6BAgaEAE) | Diptera | 2 | DNA barcoding | CRPOL054-23.COI-5P | Kühsel, 2015. Dissertation thesis. https://tuprints.ulb.tu-darmstadt.de/5214/1/Dissertation%20Sara%20K%C3%BChsel_1.pdf |
| SM | 11.07 | *Sarcophaga variegate* Scopoli | *Sarcophaga* | [Sarcophagidae](https://www.google.com/search?rlz=1C1DIMC_enIT834IT834&q=Sarcophagidae&stick=H4sIAAAAAAAAAONgVuLUz9U3MEozTMl6xGjCLfDyxz1hKe1Ja05eY1Tl4grOyC93zSvJLKkUEudig7J4pbi5ELp4FrHyBicWJecXZCSmZ6YkpgIASOM46VQAAAA&sa=X&ved=2ahUKEwiwxoPP_Nb2AhWMgP0HHRZvAcIQzIcDKAB6BAgaEAE) | Diptera | 2 | DNA barcoding | CRPOL057-23.COI-5P | Kühsel, 2015. Dissertation thesis. https://tuprints.ulb.tu-darmstadt.de/5214/1/Dissertation%20Sara%20K%C3%BChsel_1.pdf |
| SM | 11.07 | *Eupeodes* sp. | *Eupeodes* | Syrphidae | Diptera | 2 | DNA barcoding, photo | CRPOL059-23.COI-5P | Pekas et al., 2020. Biological Control 149: 104328 |
| SM | 11.07 | *Platycheirus* sp. | *Platycheirus* | Syrphidae | Diptera | 2 | DNA barcoding | CRPOL058-23.COI-5P | Gibson et al., 2006. J Appl. Ecol. 43(2):246-57 |
| SM | 11.07 | Tachinidae sp.1 | nd | Tachinidae | Diptera | nd | DNA barcoding | CRPOL060-23.COI-5P |  |
| SM | 11.07 | *Andrena* sp. | *Andrena* | Andrenidae | Hymenoptera | 2 | photo |  | Tang et al., 2019. Biol Lett 15(10):20190479 |
| SM | 11.07 | *Bombus hortorum* L. | *Bombus* | Apidae | Hymenoptera | 2 | photo |  | Wolf, Moritz, 2014. Apidologie 45(4):440-450 |
| SM | 11.07 | *Formica* sp. | *Formica* | Formicidae | Hymenoptera | 1 | photo |  | Rostás, Tautz 2010. In Seckbach & Dubinsky (eds), Springer. |
| SM | 11.07 | Formicidae | nd | Formicidae | Hymenoptera | nd | photo |  |  |
| SM | 11.07 | *Lasius emarginatus* Olivier | *Lasius* | Formicidae | Hymenoptera | 0 | photo |  | Marazzi et al., 2014. Bollettino della Società ticinese di scienze naturali 102:47-56 |
| SM | 11.07 | *Lasioglossum nitidulum* Fabricius | *Lasioglossum* | [Halictidae](https://www.google.com/search?rlz=1C1DIMC_enIT834IT834&q=Halictidae&stick=H4sIAAAAAAAAAONgVuLUz9U3MLKwKC98xGjCLfDyxz1hKe1Ja05eY1Tl4grOyC93zSvJLKkUEudig7J4pbi5ELp4FrFyeSTmZCaXZKYkpgIAHubuD1EAAAA&sa=X&ved=2ahUKEwjz2qCq_db2AhWyQvEDHTolDw4QzIcDKAB6BAgqEAE) | Hymenoptera | 2 | DNA barcoding, photo | CRPOL061-23.COI-5P; CRPOL062-23.COI-5P | Zaninotto, Dajoz, 2022. Animals 12:923 |
| SM | 11.07 | Osmiinae sp. | nd | Megachilidae | Hymenoptera | nd | photo |  |  |
| SM | 11.07 | *Ochlodes sylvanus* Esper | *Ochlodes* | [Hesperiidae](https://www.google.com/search?rlz=1C1DIMC_enIT834IT834&q=Hesperiidae&stick=H4sIAAAAAAAAAONgVuLUz9U3MKwyMs55xGjCLfDyxz1hKe1Ja05eY1Tl4grOyC93zSvJLKkUEudig7J4pbi5ELp4FrFye6QWF6QWZWamJKYCAOdub2lSAAAA&sa=X&ved=2ahUKEwi0-fvF_Nb2AhWtiv0HHeaFBwoQzIcDKAB6BAggEAE) | Lepidoptera | 2 | DNA barcoding | CRPOL055-23.COI-5P; CRPOL056-23.COI-5P | Sun et al., 2014. Annals of Botany 113(2):289-300 |
| SM | 11.07 | *Coenonympha* sp. | *Coenonympha* | Nymphalidae | Lepidoptera | 2 | photo |  | Willis, Burkill, 1903. Annals of Botany 17(2):313–50 |
| SM | 11.07 | *Thrips* sp. | *Thrips* | Thripidae | Thysanoptera | -1 | DNA barcoding | CRPOL113-23.COI-5P; CRPOL114-23.COI-5P; CRPOL115-23.COI-5P; CRPOL116-23.COI-5P; CRPOL117-23.COI-5P | Mound, Teulon, 1995. In Parker, Skinner & Lewis (eds). Springer;  Sperotto et al., 2019. Frontiers in Plant Science 10:866;  Carrillo-Arámbula et al., 2022. PLoS One 17(11): e0276865 |

**Table S2** Regression statistics of the relative abundance for Hymenoptera and Diptera families with elevation, relating to **Supplementary information** Fig. S6. Two separate regressions were carried out for active pollinators (a, c) and non-pollinators (b, d). P-values less than 0.05 are marked with an asterisk.

| **Family** | **slope** | **R^2^_Adj_** | **F** | **p** | **Regression equation** |
| --- | --- | --- | --- | --- | --- |
| a) Pollinating Hymenoptera | | | | |  |
| Andrenidae | -0.01 | 0.01 | 1.03 | 0.37 | $y=-0.01x+23.51$ |
| Apidae | 0.06 | 0.37 | 3.96 | 0.12 | $y=0.06x-48.26$ |
| Colletidae | 0.01 | -0.16 | 0.31 | 0.61 | $y=0.01x-10.33$ |
| Halictidae | -0.01 | -0.16 | 0.31 | 0.61 | $y=-0.01x+23.10$ |
| Megachilidae | -0.02 | 0.12 | 1.69 | 0.26 | $y=-0.02x+45.35$ |
| b) Non-pollinating Hymenoptera | | | | |  |
| Ichneumonidae | < 0.01 | -0.21 | 0.14 | 0.73 | $y=0.003x-2.24$ |
| Pteromalidae | -0.01 | 0.12 | 1.69 | 0.26 | $y=-0.01x+22.68$ |
| c) Pollinating Diptera | | | |  |  |
| Anthomyiidae | 0.02 | 0.68 | 11.53 | 0.03* | $y=0.02x-30.35$ |
| Calliphoridae | -0.01 | -0.16 | 0.29 | 0.62 | $y=-0.01x+16.05$ |
| Chloropidae | < 0.01 | -0.24 | 0.04 | 0.85 | $y=0.001x-0.19$ |
| Dolichopodidae | < 0.01 | -0.16 | 0.31 | 0.61 | $y=0.002x-1.93$ |
| Empididae | < 0.01 | -0.16 | 0.31 | 0.61 | $y=0.002x-1.94$ |
| Muscidae | 0.03 | 0.68 | 11.76 | 0.03* | $y=0.03x-22.23$ |
| Phoridae | 0.01 | 0.23 | 2.53 | 0.19 | $y=0.01x-13.40$ |
| Rhinophoridae | < 0.01 | -0.24 | 0.04 | 0.85 | $y=0.001x-0.19$ |
| Sarcophagidae | -0.04 | 0.67 | 10.97 | 0.03* | $y=-0.04x+87.31$ |
| Syrphidae | -0.03 | 0.76 | 17.13 | 0.01* | $y=-0.03x+68.98$ |
| d) Non-pollinating Diptera | | | | |  |
| Chloropidae | -0.01 | 0.25 | 2.66 | 0.18 | $y=-0.01x+25.75$ |
| Rhagionidae | < 0.01 | -0.11 | 0.51 | 0.52 | $y=0.004x-3.36$ |
