# Supplementary material for: Elevational shifts in reproductive ecology indicate the climate response of a model chasmophyte, Rainer’s bellflower (Campanula raineri)
Source: Ann Bot. 2024 Sep 30;135(1-2):181–98. doi: 10.1093/aob/mcae164 (PMC11805931; doi:10.1093/aob/mcae164)
Supplement: mcae164_suppl_Supplementary_Data [file mcae164_suppl_supplementary_data.zip › aob-24018-s03.docx]

Type of Article: Original Article

Title: Elevational shifts in reproductive ecology indicate the climate response of a model chasmophyte, Rainer’s bellflower (*Campanula raineri*)

**Names of the authors:**

Sara Villa^1,2^, Giulia Magoga^3^, Matteo Montagna^3,4^_,_ Simon Pierce^2,^*

**Running title:** Elevation/climate effects on chasmophyte reproduction

**Affiliation of the authors:**

^1^ Institute for Sustainable Plant Protection, National Research Council, via Madonna del Piano 10, 50019, Sesto Fiorentino, Italy

^2^ Department of Agricultural and Environmental Sciences - Production, Landscape, Agroenergy (DiSAA), University of Milan, via G. Celoria 2, 20133, Milan, Italy

^3^ Department of Agricultural Sciences, University of Naples “Federico II”, via Università 100, 80055, Portici, Italy

^4^ BAT Center ‑ Interuniversity Center for Studies on Bioinspired Agro‑Environmental Technology, University of Napoli “Federico II”, via Università 100, 80055, Portici, Italy

*** Corresponding author**

Simon Pierce: simon.pierce@unimi.it, Department of Agricultural and Environmental Sciences - Production, Landscape, Agroenergy (DiSAA), University of Milan, via G. Celoria 2, 20133, Milan, Italy. tel. +39 0250316785

ORCID ID Sara Villa: 0000-0002-9238-9839; e-mail: sara.villa@ipsp.cnr.it

ORCID ID Giulia Magoga: 0000-0002-0662-5840; e-mail: giulia.magoga@unina.it

ORCID ID Matteo Montagna: 0000-0003-4465-7146; e-mail: matteo.montagna@unina.it

ORCID ID Simon Pierce: 0000-0003-1182-987; e-mail: simon.pierce@unimi.it

**Abstract**

- ***Background and aims*.** Elevation gradients provide ‘natural experiments’ for investigating plant climate change responses, advantageous for the study of protected species and life forms for which transplantation experiments are illegal or unfeasible, such as chasmophytes with perennial rhizomes pervading rock fissures. Elevational climatic differences impact mountain plant reproductive traits (pollen and seed quality, sexual *vs.* vegetative investment) and pollinator community composition; we investigated the reproductive ecology of a model chasmophyte, *Campanula raineri* Perp. (Campanulaceae), throughout its current elevational/climatic range to understand where sub-optimal conditions jeopardise survival. We hypothesised that: 1) reproductive fitness measures are positively correlated with elevation, indicative of the relationship between fitness and climate; 2) *C. raineri*, like other campanulas, is pollinated mainly by Hymenoptera; 3) potential pollinators shift with elevation.
- ***Methods.*** We measured pollen and seed quality, seed production, the relative investment in sexual *vs*. vegetative structures and vegetative (Grime’s CSR) strategies at different elevations. Potential pollinators were assessed by combining molecular and morphological identification.
- ***Key results.*** Whereas CSR strategies were not linked to elevation, pollen and seed quality were positively correlated, as was seed production per fruit (Hypothesis 1 is supported). The main pollinators of *C. raineri* were Apidae, Andrenidae, Halictidae (Hymenoptera) and Syrphidae (Diptera), probably complemented by a range of occasional pollinators and visitors (Hypothesis 2 partially supported). Potential pollinator communities showed a taxonomic shift towards Diptera with elevation (particularly Anthomyiidae and Muscidae) and away from Hymenoptera (Hypothesis 3 was supported).
- ***Conclusions.*** Pollinator availability is maintained at all elevations by taxa replacement. However, reduced pollen quality and seed production at lower elevations suggest an impact of climate change on reproduction (especially <1,200 m a.s.l., where seed germination was limited). Aside from guiding targeted conservation actions for *C. raineri*, our results highlight problems that may be common to mountain chasmophytes worldwide.

**Keywords:** Adaptive strategy, altitudinal gradient, *Campanula raineri* Perp., climate change, chasmophytes, COI DNA barcoding, germination, insect pollinators, mountain species, population ecology, reproductive fitness, species conservation.

**Introduction**

Elevation gradients, exhibiting strong climatic changes over relatively short distances, provide natural ‘space-for-time’ experiments, a well-established methodology for investigating the responses of wild plants to climate change (Körner, 2007; Tito *et al.*, 2020). Observation of wild plants along elevation gradients is also useful for providing baseline data in the broader context of ecological relationships, such as plant-pollinator associations. Species ranges are generally contracting and moving uphill in response to climate warming, at a rate that is taxon or life form specific. Some groups, particularly rare alpine specialists are more sensitive and responsive than others, and the differential migration rates uncouple the biotic components of ecosystems (*e.g.*, Rumpf *et al.*, 2018; Zu *et al.*, 2021; Geppert *et al.,* 2023).

Observation of wild plants over an elevation gradient is also useful because experimental investigation is not feasible for all life-forms. For instance, while reciprocal transplant experiments can be used to account for the genetic and ecotypic adaptation of populations to local climate, such an approach usually involves relocation of intact turfs to maintain the plant community context of the study species (*e.g.*, Cui *et al.* 2018; Khedim *et al.* 2023), and is not amenable to all plant species. Mountain chasmophytes (fissure-dwelling rock-face species), as perennial, rhizomatous plants growing within rock crevices, are not suited to extraction nor to transport of the plant rooted within its substrate. They are typically already limited to mountaintops and, due to exposure and a lack of contact with soil resources, are particularly prone to climate oscillations (Dolezal *et al.*, 2020; Inouye, 2020). Paradoxically, this life-form encompasses a range of rare and legally protected species (protection that specifically outlaws manipulation) which are precisely the species for which understanding climate responses is most urgently required yet most difficult to obtain (*e.g.*, Casazza *et al.*, 2018). Another approach, cultivating juveniles under standardised conditions in ‘reciprocal transplant gardens’, can identify ecotypic differences (*e.g.*, Johnson *et al.*, 2022) but cannot emulate the growth responses of adult perennials established within natural rock crevices. Despite the exigent need to understand chasmophyte responses, to date the study of chasmophyte/climate relationships (*e.g.*, for the *Campanula lehmanniana* complex; Nobis *et al.* 2023) has relied on abiotic niche modelling without direct observation of ecological variability or biotic interactions *in situ*.

How elevation influences the inherent reproductive capabilities of plants has been investigated, but an integrated view spanning the capacities of the plant and wider ecological relationships along elevation gradients is absent. In general, responses involve relative biomass allocation to sexual (*vs.* vegetative) structures, flower longevity and stigmatic receptivity generally increasing with elevation (*e.g.*  chasmophytic *Campanula* spp.; Bingham and Orthner, 1998; Blionis and Vokou, 2001). Moreover, vegetative, clonal reproduction is a common way of reducing risk to delicate flowers (Weppler *et al.*, 2006; Arroyo *et al.*, 2017; Körner, 2021), also allowing colonisation of disturbed habitats such as screes (Evette *et al.*, 2009). At lower elevations, higher temperature tends to limit pollen tube growth, ovule fertilisation and fruit and seed set (Flores-Rentería *et al.*, 2018). Conversely, low temperatures can reduce pollen germination, fertilisation success, seed maturation and survival (Totland, 2021), and plants at the highest elevations can suffer pollen limitation (*e.g.*, Jiang and Xie, 2020). Species adapted to higher elevations exhibit a seed dormancy phase interrupted by environmental stimuli (changes in temperature or light regimes; Fernández-Pascual *et al.*, 2021) and lower temperature optima for seed germination (Vera, 1997; Yucedag *et al.*, 2021). Thus, reproductive fitness is a central issue for plant climate responses and appears to be maximal under species specific or life-form specific optimal conditions. This can be further complicated by population size effects, whereby smaller populations, particularly at the edge of the distributional range, are prone to inbreeding depression (Allee effects; Allee *et al.*, 1949; see Dawson-Glass and Hargreaves, 2022).

Aside from the inherent capabilities of the plant, ecological interactions, particularly those linked to reproduction, may also change with elevation. The dependence of pollinator abundance and activity on temperature is well studied (*e.g.* Bingham and Orthner, 1998; Arroyo *et al.*, 2006, 2017; Lara-Romero *et al.*, 2019), and is known to generally affect the availability, diversity and activity of the pollinator fauna, typically with Hymenoptera and Lepidoptera progressively replaced by Diptera in cooler, moister conditions (Warren and Harper, 1988; Lefebvre *et al.*, 2018; Minachilis *et al.*, 2020; McCabe and Cobb, 2021). Bumblebees are the most common hymenopteran pollinators at high elevation, active at relatively low temperatures and higher wind speeds (Bergmann *et al.*, 1996). Furthermore, high-altitude flower visitors are generally less selective in foraging choice, with erratic visitation patterns: this buffers pollination networks against local extinctions, favouring community stability (Arroyo *et al.*, 2006; Ramos-Jiliberto *et al.*, 2009; Chesshire *et al.*, 2021).

In the present study, Rainer’s bellflower (*Campanula raineri* Perp., Campanulaceae), endemic to a limited range in the Northern Italian Alps (Fig. 1), is used both as a model alpine chasmophyte and as an example for investigating the fitness and ecology of a rare, protected species along an elevation gradient. Elevation, as a proxy for climate, appears to be a principal factor affecting survival for *C. raineri* because the species recently became extinct at the lowest elevation site (Monte Barro, Lecco; 922 m a.s.l.) despite the site being a regional park actively managed for conservation purposes since 1983. Here, *C. raineri*, other chasmophytes (*e.g.*  *Physoplexis comosa* (L.) Schur*., Primula glaucescens* Moretti), chasmophyte habitats and neighbouring grassland habitats have been specific conservation targets of an EU Life project (LIFE00NAT/IT/007258) involving management and population reinforcement activities. Local extinction despite this active conservation and mitigation of land use change over the past forty years is a strong indicator that factors operating beyond the control of the park have been decisive, the most plausible culprit being climate change. Variables such as competition from larger plants exacerbated by nitrogen deposition are unlikely to be issues for chasmophytes isolated in fissures of calcareous rock, where local extinction is a process of simple loss rather than replacement by other species. Another reason for focussing on elevation during the present study is that the experimental manipulation of *C. raineri*, as for many rare chasmophytes, would entail a scale of disturbance that is literally illegal (in this case, the species is listed as an Annex C1 “*species in need of rigorous protection*” by Lombardy Regional Law No. 10 of the 31 March 2008). The species is also relatively enigmatic: for instance, the pollinators of *C. raineri* have not been identified (aside from *Apis mellifera* L., found carrying pollen at one site; Galimberti *et al.*, 2014). *Campanula* species are typically pollinated by Hymenoptera; in particular solitary bees (Megachilidae, Andrenidae) and bumblebees (*Bombus* spp., Apidae) (Inoue *et al.*, 1996; Milet-Pinheiro *et al.*, 2015; 2021; D’Antraccoli *et al.*, 2019; Villa, 2023). Certain pollinators show a predilection for the genus *Campanula* (*i.e.* *Chelostoma campanularum* Kirby, *C. rapunculi* Lepeletier*, Hoplitis mitis* Nylander; Megachilidae) (*e.g.*  Schlindwein *et al.*, 2005; Milet-Pinheiro *et al.*, 2013; 2015; 2021), being more sensitive than polylectic species (generalist pollinators) to specific constituents of *Campanula* floral scents (Milet-Pinheiro *et al.*, 2013; 2015; Brandt *et al.*, 2017). These relationships are likely to change with elevation: on Mount Olympus (Greece), Megachilidae and Andrenidae are the main visitors of *Campanula* at lower elevations, while bumblebees and Melittidae become the primary pollinators above 1,850 m a.s.l. (Blionis and Vokou, 2001), agreeing with similar results in the Swiss Alps and the Rocky Mountains (Cresswell and Robertson, 1994; Bingham and Orther, 1998). Other Hymenoptera (*e.g.*  *Apis* *mellifera* L. and *Xylocopa* spp., Apidae; *Lasioglossum* spp., Halictidae) and different species of Diptera (mainly Syrphidae and Muscidae) are also reported as possible pollinators of the genus *Campanula* (Janzon, 1983; Eisto *et al.*, 2000; Blionis and Vokou, 2001; D’Antraccoli *et al.*, 2019; see also Janzon, 1983; Kozuharova *et al.*, 2005). Thus a range of potential pollinators and shifts in the pollinator community are potentially linked to elevation for this species, although this is currently not known with any degree of certainty.

Here, our aim is to understand the relationship between elevation, functioning and wider reproductive ecology of this species, as a model chasmophyte and an example of a species that is so rare that it can only realistically be investigated via observation *in situ*. Specifically, based on the literature regarding similar species, we hypothesized that: (1) elevation is positively correlated with reproductive traits, such as pollen and seed quality (considered here in terms of viability, germination, seed mass) and investment in sexual *vs*. vegetative effort (with plants retaining capacity for clonal reproduction, as observed in other mountain species with showy flowers); (2) *C. raineri* is a pollination specialist (*i.e.* has specific pollinators): the broadly campanulate flowers favour pollination by Hymenoptera, and in particular solitary bees (Megachilidae, Andrenidae) or bumblebees (*Bombus* spp.); (3) *C. raineri* pollinators change with elevation (specifically: at high elevations bumblebees replace solitary bees and the contribution of Diptera to pollination also increases).

**Materials and Methods**

***Data collection***

*Study species*

Rainer’s bellflower *(Campanula raineri*; Campanulaceae) is a perennial species endemic to the Italian calcareous Prealps (Lombardy and Trentino Alto Adige/Südtirol regions, northern Italy), with scattered populations throughout an area not exceeding 8,000 km^2^, between 1,000-2,200 m a.s.l. (Aeschimann *et al.*, 2004; Pignatti, 2018; www.biodiversita.lombardia.it/sito/). With regard to life-form, rosettes produce buds at the level of the substrate (*i.e.* a [hemicryptophyte](https://context.reverso.net/traduzione/inglese-italiano/scapose+hemicryptophytes); *sensu* Raunkiær, 1934), and can represent ramets of more extensive genets (*sensu* Harper and White’s (1974) interpretation of clonal growth, in which seeds give rise to genetic individuals or genets that develop and spread through reiterated modular units or ramets), with rhizomes following rock crevices or springing up from debris and screes (Pignatti, 2018; Körner, 2021). Indeed, while the species is a sexually reproducing flowering plant, clonal growth via rhizomes allows it to form extensive vegetative colonies with each rosette/ramet is essentially a vegetative clone that is capable of flowering. Although plants of *C. raineri* are small, not exceeding 10 cm in height, the blue-violet bell-shaped flowers are disproportionately large, at around 3-4 Ø × 3 cm (Pignatti, 2018). These features make *C. raineri* particularly showy during the flowering period (July-August) providing a crucial pollination advantage in barren, rocky environments (Billings and Mooney, 1968; Bliss, 1971; Körner, 2021). The species (along with all Campanulaceae) exhibits secondary pollen presentation, or the exhibition of pollen by the style. Pollen display occurs before stigma ripening (Erbar and Leins, 1995; Vranken *et al.*, 2014; Crowl *et al.*, 2016), ensuring a staggered male and female phase during anthesis (protandry) which limits self-pollination (Nyman, 1992). In the Campanuloideae, the pollen deposition mechanism all around the style is probably linked with a return to floral actinomorphy, and both traits promote pollen collection regardless of the angle at which the pollinator approaches the flower (Neal *et al.*, 1998; Crowl *et al.*, 2016), encouraging pollination by generalist taxa such as bees and flies (*i.e.*, a general entomophilous pollination syndrome).

Regarding the choice of *C. raineri* as a study species, populations at sites with historical records and below 1,000 m a.s.l. appear to have become extinct in recent decades (*e.g.* on Monte Barro, Lombardy, Italy, or Monte Generoso, Canton Ticino, Switzerland/Lombardy, Italy; Arietti and Fenaroli, 1963; Brusa, 2005; S.V. and S.P. personal observations).

Collection of a limited amount of leaf and reproductive material, as detailed in a project proposal submitted to the regional government, was permitted under the auspices of Decree number 9336, Act 855, *sensu* article 8 of Regional Law 10/2008, conferred on researchers Simon Pierce and Sara Villa by the General Direction of Environment and Climate of the Lombardy Regional Government on the 08/07/2021. This permit did not allow the collection of whole plants or damage to the habitat or substrate.

*Reproductive traits of* C. raineri

The reproductive effort of *C. raineri* across populations was investigated using a variety of traits measured in the field or with experimental tests (see Fig. 1 for the map of sampling sites and Table 1 for details including population locations and measured traits). To investigate whether the reproductive investment (sexual *vs.* vegetative clonal growth) changes with elevation, we considered the ratio between the number of flowers and the total number of reproducing structures (*i.e.* flowers/[flowers + rosettes]) per genet. The flowers:reproducing structures ratio (FRR) was estimated by field counts in the following localities (representing the entire elevational gradient of the species): Sasso Malascarpa, Corni di Canzo, Grigna Meridionale, Piani di Artavaggio, Pizzo della Presolana, Monte Cavallo (Lombardy Region). Counting was performed on a minimum of 3 genets (Piani di Artavaggio) and a maximum of 73 (Monte Resegone; Table 1B). Note that the population of Piani di Artavaggio consists of extensive and intertwined genets (often with hundreds of rosettes) that are difficult to delimit; at least three spatially distinct genets were identified in the area considered for the counts, but the possibility remains that the actual number of individuals could be much higher. Counting of genets was performed for representative areas of the target populations to estimate the population density (PD; number of genets per unit ground area). Counting was carried out in accessible and environmentally homogeneous areas (10×10 m^2^). Population size within the entire area covered during sampling was also estimated (Table 1B). Due to the widely varying extent of the habitat suitable for the growth of *C. raineri* at the sampling sites, only the values reported for the Sasso Malascarpa and Corni di Canzo localities are direct counts of actual population size, while for the other sites only part of the area occupied by the species was covered, and reported values should be considered estimates of the minimum population size.

The extent of competitivity (C), stress-tolerance (S) and ruderality (R) *sensu* Universal Adaptive Strategy Theory (UAST; Grime 1974; 1977; 2001; Grime and Pierce, 2012) was calculated for the same six populations to investigate intra-specific functional variation. According to UAST (Grime and Pierce 2012) viable suites of functional (adaptive) traits have evolved in response to either C-selection (consistently productive niches select for traits maximising resource acquisition and resource control, involving rapid attainment of large individual size), S-selection (abiotically variable and unproductive niches select for conservative growth, longevity and traits maintaining metabolic performance of the individual), or R-selection (frequent lethal disturbance events selecting for rapid growth and early completion of the lifecycle at small size). For plant CSR classification, we collected a total of ten mature leaves from as many randomly selected genets (one leaf each) for each population during the period of maximum vegetative development (July/August 2021), these were wrapped in moistened paper towels and aluminium foil, transported in an insulated cool bag and stored at 4°C overnight to attain turgidity. Leaf fresh weight (LFW; mg) and leaf area (LA; mm^2^) measurements were taken the next morning, while leaf dry weight (LDW; mg) was measured after 24 h at 60°C. Proportional measures of C, S, and R were then calculated with the ‘StrateFy’ CSR classification tool (for detailed explanation of method, see Pierce *et al.*, 2017). In summary, the Pierce *et al.* (2017) CSR-classification method compares leaf area (LA; mm^2^), specific leaf area (SLA; photosynthetic tissue density denoted by LA divided by LDW) and leaf dry matter content (LDMC; calculated as LDW/LFW×100), which are positively correlated, respectively, with the three main extremes of the global spectrum of plant form and function: organ/plant size, acquisitive resource economics and conservative economics (Díaz *et al.* 2016). In practice, this involves the statistical comparison, for each leaf sample, of the trade-off between these traits (*i.e.* relative position along a spectrum of variation) against the trade-off evident in the world vascular plant flora, quantifying the absolute extent of size variation and position along the acquisitive/conservative spectrum, and assigning proportion values for these adaptive endpoints (a Microsoft Excel spreadsheet including these algorithms is available as Supplementary Material from: https://besjournals.onlinelibrary.wiley.com/doi/full/10.1111/1365-2435.12722). For instance, a C:S:R ratio of 10:70:20 % indicates a relatively large extent of stress-tolerance, or conservative functioning, but some ruderality and lesser competitive ability. The three-way trade-off between C, S and R values was presented using the ternary plotting function of Sigmaplot.

**Pollen quality**. Pollen quality was investigated in terms of pollen viability (PV) and pollen germination (PG). For both purposes, a total of 4 or 5 pistils were collected from each of six populations (Corni di Canzo [4 pistils], Sasso Malascarpa, Grigna Meridionale, Piani di Artavaggio, Pizzo della Presolana, Monte Cavallo [5 pistils each]; Fig. 1, Table 1) between July and August 2021 from recently blooming flowers of different plants (during the male phase) to maximise pollen quality and yield (Nyman, 1992). Sampling was performed in the morning (~09:00) to ensure the collection of fresh pollen. To prevent the development of mould, pistils were stored in a 1.5 ml tube, covered with cotton and a small amount of silica gel beads, transported in an insulated cool bag and then stored at 4°C overnight before laboratory treatment.

To measure PV, pollen grains scraped from the style were placed on a microscope slide with a drop of 1% Triphenyl-tetrazolium chloride (TTC) solution (0.02 g TTC and 1.2 g sucrose in 2 ml of distilled water; Sulusoglu *et al.*, 2014). Depending on the amount of pollen, 4 to 10 slides were prepared for each population, covered with a coverglass and stored in darkness for two hours. PV was observed with darkfield illumination using a compound microscope (Zeiss Axio Zoom V16; Oberkochen, Germany), and counts of viable and unviable grains were made from images randomly acquired (covering about the 20% of each slide) with a digital camera (Zeiss Axiocam 506). Counting of viable pollen grains was performed on a minimum of 5,866 and a maximum of 23,735 grains per population (Table 1B). Pollen grains dyed orange or bright red were considered viable (Sulusoglu *et al.*, 2014).

To measure PG, pollen grains were sown on a sterile agar medium (7 g L^-1^) enriched with sucrose (150 g L^-1^), CaCl_2_(H_2_O) (152 mg L^-1^) to satisfy the calcium requirements of cells, and H_3_BO_3_ to stimulate pollen tube growth (Sulusoglu *et al.,* 2014). The pH was adjusted to 5.7 and the medium was autoclaved at 121°C and 101,325 Pa for 20 minutes, and poured into 6 cm diameter Petri dishes in sterile conditions. The pollen grains were sown on two replicate Petri dishes per pistil (for a total of 8/10 dishes per population), using a steel dissecting hook to scrape pollen from the style. After incubation for 24 h at room temperature (~25°C), germinated pollen grains were observed under the microscope, and counted from images randomly acquired (covering about the 5% of each Petri dish) with the digital camera. Counting of germinated pollen grains was made on a minimum of 2245 and a maximum of 7092 grains per population (Table 1B). Grains were considered to be germinated when the pollen tube length reached the diameter of the grain (Sulusoglu *et al.*, 2014). For each replicate, the proportion of viable or germinated pollen grains was calculated.

**Seed quality.** Seed quality was investigated in terms of mass, seed viability (SV) and seed germination (SG). Seeds were collected in September 2020 from nine populations (Corni di Canzo, Sasso Malascarpa, Grigna Meridionale, Piani di Artavaggio, Monte Resegone, Monte Venturosa, Pizzo della Presolana, Pizzo Arera, Monte Cavallo; Fig. 1, Table 1), cleaned and stored at 15% relative humidity and external ambient temperature until laboratory treatments (sowing and viability tests), to ensure a cold stratification period and break the dormancy phase (Villa *et al.* 2021). Before all laboratory analyses, seeds collected from each individual were weighed to estimate the total seed mass per fruit (TSMF; total seed mass:number of collected fruits). Then, seeds from each population were merged and 10 subsamples of 750 seeds weighed to calculate the mean weight of a single seed (single seed mass; SSM) for each population. Subsequent measurements and laboratory analyses were performed sampling from pooled seeds. Finally, the number of seeds per fruit (NSF) was estimated first calculating the number of seeds collected from each individual (mass of the seeds collected from each individual divided by SSM) and then dividing it by the number of collected fruits Table 1B).

Ten months after collection (July 2021), SV was checked using a standard tetrazolium test. Counting of viable seeds was conducted on a minimum of 38 and a maximum of 55 seeds per population (Table 1B). Seeds were rehydrated with distilled water for 12 hours, scarified in a 5% sodium hypochlorite solution for 5 minutes, and then rinsed 6 times with distilled water (Hsiao *et al.*, 1979; AOSA/SCST, 2010). Seeds were dipped in 1% solution of 2,3,5-triphenyl tetrazolium chloride (TTC; 0.02 g of TTC in 2 ml of distilled water), left to react at 35°C for 8 h in darkness and finally stored overnight at 4°C in darkness. Viability of all treated seeds was observed under the compound microscope, and counts were performed from digital images. Seeds dyed red were considered viable (AOSA/SCST, 2010), and the proportion of viable seeds was calculated for each population.

SG was measured through *in-vitro* sowing and germination counts, as detailed in Villa *et al.* (2021). Approximately six months after collection, seeds were sterilised in 10% NaOCl solution and sowed in sterile agar medium (6 g L^-1^) enriched with sucrose (20 g L^-1^), Murashige and Skoog (1962) mineral salts (2.2 g L^-1^), activated charcoal powder (0.5 g L^-1^) and gibberellic acid (GA3; 40 mg L^-1^). Ten replicates with approximately 25 seeds each were made for each population. Sown seeds were incubated in a growth chamber alternating 16 h of light at 20 °C and 8 h of dark at 10 °C for 28 days. Germination was weekly monitored by cumulative counting, while samples with mould development were discarded. After removing any mould-contaminated Petri dishes, counting of germinated seeds was made on a minimum of 174 and a maximum of 548 seeds per population (Table 1B). The proportion of germinated seeds was calculated for each replicate. Mean germination percentages of each population over time and variability among replicates were visualised using the *drc* (Ritz *et al.*, 2015), *nlme* (Pinheiro *et al.*, 2000; 2023) and *ggplot2* (Wickham, 2016) packages in the R environment. In cumulative germination curves, the angular coefficient at the inflection point was used to compare germination speed among populations.

*Visitor and pollinator assessment*

The insect fauna associated with *C. raineri* flowers was investigated at six sampling sites across the elevational range: Sasso Malascarpa, Corni di Canzo, Grigna Meridionale, Piani di Artavaggio, Pizzo della Presolana, Monte Cavallo (Fig. 1, Table S1). We combined two different methods to identify potential pollinators (*i.e.* direct observation and molecular identification of insect fauna collected *in situ*). The inaccessibility of the sites where *C. raineri* grows renders classical methods particularly challenging (*i.e.* direct observations/captures and passive tools such as Malaise traps, flight intercept traps, pan traps; Cane *et al.*, 2000). Moreover, combining different strategies allowed moderation of the flaws inherent to each method, and provided a more comprehensive view. In this study, collection permits were not required for target taxa, as they are not included in the annexes of Habitat Directive 92/43/EEC nor in the list of species of regional interest as per Lombardy regional law 10/2008 (D.g.r. 7736/2008). Moreover, no special ethical permission was required for the taxa collected (Directive 2010/63/EU).

**Sampling and molecular identification of pollinators.** Insects in the vicinity of flowers were collected during sunny, not windy days with flight interception sticky traps, placed adherent to the substrate. Two traps (10×10 cm) were placed at each sampling point, one coloured violet to simulate flowers and one left blank to check background visitation. According to *C. raineri* population size, one or two sampling points were set at each site, with sticky traps placed in correspondence of plants with at least five open flowers. One sampling point was set at Sasso Malascarpa and Monte Cavallo, two at the remaining sites. Sticky traps were placed at around 09:00 and removed after ~8 hours (one-day sampling). Additionally, arthropods stationed on the flowers were collected manually. Captured arthropods were placed in 96% ethanol and stored at -30°C until DNA extraction.

A preliminary morphotype classification was performed under a stereomicroscope (Leica MS5; Wetzlar, Germany). DNA was extracted from one representative specimen per morphotype (leg tissue for the largest specimens, whole body for the smallest). DNA extraction followed a previously published method (Mereghetti *et al.*, 2019) with the following modifications: tissues were crushed using a sterile pestle and mortar, proteinase K incubation time and temperature were reduced to 2.5 hours and 37°C, respectively; DNA precipitation with isopropanol was carried out overnight; the pellet was eluted in 10 μL of water. The 5’ region of the mitochondrial Cytochrome c oxidase subunit I (COI) gene was amplified by PCR using the universal barcode primers LCO1490/HCO2198 (Hebert *et al.*, 2003; Boheme *et al.*, 2012). Amplification of 100-200 ng of DNA template was performed in 25μL of reaction mixture (Magoga *et al.*, 2018), following the thermal conditions reported by Montagna *et al.* (2013). Successful amplifications were checked by 1.5% agarose gel electrophoresis. PCR products were sequenced in one strand using the forward primer LCO1490 using the Sanger method by Microsynth Seqlab GmbH (Göttingen, Germany).

Sequences were quality-controlled using Geneious R8 (Biomatters Ltd., Auckland, New Zealand; license owner, M.M.) and aligned using the MUSCLE algorithm (Edgar, 2004) implemented in MEGA 11.0.10 (Kumar *et al.*, 2018). Molecular identification was performed after checking for the presence of open reading frames using EMBOSS Transeq (www.ebi.ac.uk/Tools/st/emboss_transeq). Clean nucleotide sequences were compared with reference sequences on online databases GenBank (Benson *et al.*, 2013) and Barcode of Life Data Systems BOLD (Ratnasingham and Hebert, 2007) using the BLAST tool (Altschul *et al.*, 1990) and the BOLD identification tool, respectively (last accessed, October 2022). Species level identification was assigned for sequence identities/similarities ≥ 98% between query and reference sequences. Determination to genus and family level was assigned for sequence similarities of 94-98%, and <94%, respectively (Boehme *et al.*, 2012; Elbrecht and Leese, 2015). The geographic distribution of each taxon was checked using the Global Biodiversity Information Facilities database (GBIF; https://www.gbif.org/) in order to assess its presence in the study area. Sequences were submitted to the BOLD system (https://www.boldsystems.org/; see **Supplementary Information** Table S1 for BOLD IDs).

**Observation and morphological identification of flower visitors.** During the collection of arthropods with sticky traps, flowers at sampling points were monitored for 30 min three times a day, with one observation in the morning (09:00), one observation around noon and the last observation in the late afternoon (17:00), in order to include the main periods of activity of the principal pollinators (*e.g.*  Bjerge *et al.*, 2022). Images of visiting insects were taken through continuous shooting (10 photographs per sec) using a digital camera (Olympus SZ-14; Tokyo, Japan). Morphological identification from images was performed, when possible, with the support of experts. In the subsequent analyses, data from any multiple replicates (*i.e.* possible different sampling points) and different times (morning, noon and late afternoon) at the same site were merged.

Presence data collected by the two methods were merged, and subsequent data analyses were carried out only on taxa identified at least to the genus level, with the only exception of Mantel tests and the regression of the relative abundance of orders against elevation (see below), in which a lower level of detail was allowed. Where attribution to species level was uncertain, only the genus was retained (*e.g.*  *Andrena* sp.). Where there was no certainty that two taxa represented the same species, the distinction into different species was maintained, as for example in the case of *Bombus,* for which *Bombus* sp.1, *Bombus* sp.2, *Bombus* sp.3 were assigned, in addition to *Bombus hortorum*L. The known ecological role of each taxon was checked by consulting specific literature [reported in **Supplementary Information** Table S1], and indicated by a number, distinguishing 4 main categories: pollinators (2), occasional pollinators (1), phytophagous (-1) and neither pollinator nor phytophagous taxa (neutral interaction with *C. raineri*, 0). Although *Thrips* spp. are occasionally pollinators for Ericaceae (García‐Fayos and Goldarazena, 2008; Eliyahu *et al.*, 2015), in the present study they were considered phytophagous species, based on field observations by S.V. and bibliographical support (*e.g.*  Mound and Teulon, 1995; Sperotto *et al.*, 2019).

***Statistical analyses***

*Reproductive traits of* C. raineri

**Multivariate analysis***.* For each variable for which replicate measures were taken (FRR, CSR score, PV, PG, SSM, TSMF, SG), the mean value and standard error were calculated for each population (Table 1)*.* We first performed a principal component analysis (PCA) using, as input variables, the mean values of elevation, FRR, PD, PG, PV, SSM, TSMF, SG, SV, the C, S and R scores, and Wright’s inbreeding coefficient (F_IS_) for each population. This latter parameter was estimated in a parallel population genomic study based on a 2b-RAD approach (Villa, 2023) and included here as an indicator of possible Allee effects. PCA was performed on a standardised dataset in the R environment using the InDaPCA function (Podani *et al.*, 2021) and the *BAT* package (Cardoso *et al.*, 2015). PCA loadings were rescaled by a factor of 0.3 for clearer visualisation. Moreover, a correlogram was constructed on the same dataset used for the PCA, to explore the significance of each pairwise correlation. After normality testing (Shapiro test), the correlogram was built with the *pairs.panels* function of the R *psych* package (Revelle, 2022), using default settings (Pearson’s correlation), and the significance of each correlation was tested.

**Analysis of single traits.** To explore in more detail the response of populations with regard to specific traits, univariate analyses (analysis of variance - ANOVA, and linear regressions with elevation) were performed including the replicates measured at each site, as detailed below. The choice of variables to be further investigated (CSR scores, FRR, PV, PG, SV, SG, SSM, TSMF, NSF) was based on both the degree of significance of the exploratory multivariate analyses and the relevance of the characters to the working hypotheses. Specifically, traits with significant correlations with elevation as shown by the correlogram, and traits with PC1 (PCA) loadings ≤ -0.70 (PV, PG, SV, SG) were investigated further. Seed mass, production and FRR were also analysed despite not exhibiting significant relationships with elevation, as these are key traits in reproductive fitness studies. Also, CSR-score variation was examined as an indicator of variability in vegetative functioning and local effects on population adaptation, to complement information on reproductive functioning. ANOVA was applied for exploratory analyses to reveal inter-population variation not necessarily linked to elevation (and our main hypotheses) such as relationships between, for example, population size and seed mass, or sources of disturbance and CSR-scores. As these are not directly related to the study hypotheses but may be generally pertinent, the results of these extra analyses are available in the supplementary material.

Regarding variation in vegetative strategies (CSR strategies), an ANOVA followed by Tukey’s multiple comparison post-hoc procedure was performed (SYSTAT 12; SPSS, Illinois, USA) to compare the mean R scores between populations. Only R selection was tested because R selection was the prevalent strategy and represented the main direction of variability (as demonstrated by Fig. S1), with C scores showing constant values among populations and the extent of S effectively the inverse of R selection in this case (Table 1A). Also, SSM in different sampling locations was compared using an ANOVA, followed by Tukey’s multiple comparison procedure. Analyses were performed with the R packages *plyr* and *agricolae* and results visualised with *ggplot2* (Wickham, 2011; 2016; de Mendiburu and Yaseen, 2020).

After verifying model assumptions, regressions against elevation were performed for the following single traits: FRR, PV, PG, SV, SG, SSM, TSMF, NSF. With regard to the traits expressed as ratios or proportions (*i.e.* FRR, PV, PG, SG and SV) logistic regressions were applied, with dependent variables for each regression being the two original measures of each trait, rather than the proportion. For example, for FRR the dependent variables were the number of flowers and the number of rosettes; for PG the number of germinated and non-germinated pollen grains being the dependents. Regressions for traits expressed as absolute measures (*i.e.*, SSM, TSMF and NSF) were performed applying a simple linear model. Logistic and linear regressions were performed using the R package *stats* (R Core Team, 2022); for SSM the regression was performed using the package *robustbase* to ensure robustness of fit (Maechler *et al.*, 2023).

*Insect communities*

Differences in insect communities across sites at different elevations were tested with the analysis of similarities (ANOSIM) to verify the impact of elevation on taxonomic composition (Clarke and Green, 1988; Borcard *et al.*, 2011; Oksanen *et al.*, 2017). Different analyses were performed on taxa presence/absence data, also considering the different ecological roles. The analyses were thus performed on different subsets of the taxa table [**Supplementary information** Table S1], specifically considering i) the entire dataset (comprising all the ecological roles), ii) pollinators *sensu stricto* (ecological role 2), iii) pollinators *sensu lato* (ecological roles 1 and 2), and iv) phytophagous and neutral species (ecological roles -1 and 0). ANOSIM tests require replicates, and were thus performed grouping sampling localities and their communities into pairs as follows: Sasso Malascarpa and Corni di Canzo: low altitude (below 1,500 m a.s.l.); Grigna Meridionale and Piani di Artavaggio: intermediate altitude (between 1,500 and 1,800 m a.s.l.); Pizzo della Presolana and Monte Cavallo: high altitude (above 1,800 m a.s.l.). Mantel tests were also performed to assess whether the proportion of Diptera, Hymenoptera and Lepidoptera (the orders potentially including the main pollinators of *C. raineri*) changed with elevation, thereby testing the existence of a taxonomic shift along the elevation gradient. Relative abundance (of Orders or Families) was calculated as the ratio between all taxa of the target Order (or Family) with respect to the total number of detected taxa at each site, which was considered as the response matrix (after applying Manhattan distance), and elevation was set as an explanatory variable (after applying Euclidean distance). Finally, single regressions of the relative abundance of detected orders and Diptera and Hymenoptera families (the most abundant and relevant to the study) with elevation were performed.

**Results**

***Reproductive traits of* C. raineri**

In preparation for the multivariate analysis, data for separate parameters were collated (Table 1) and a description of the data obtained is summarized here. Population density ranged from 0.01 to 0.73 individuals m^-2^ (Pizzo della Presolana and Grigna Meridionale, respectively), population size being lowest for Sasso Malascarpa, with approximately 30 individuals. Minimum estimates for the other populations suggested a number of individuals at least double this, and up to several hundreds. With regard to ecological strategies (CSR strategies) plants from all study populations exhibited extensively R-selected characteristics (76% to 90% ruderalism or R-selection, in Grigna Meridionale and Pizzo della Presolana, respectively), while C scores ranged from 6 to 9%, and S scores ranged from 3% to 19% (Fig. S1). F_IS_ remained negative and very close to zero in all populations, with values ranging between -6.8 and -4.3 × 10^-4^ (Table 1A). With regard to pollen quality, the mean proportion of viable pollen grains was lowest for the Sasso Malascarpa population (44.7%) and highest for the population of Monte Cavallo (93.6%). The mean proportion of germinated pollen grains was lowest for the Sasso Malascarpa population (36.1%) and highest for the Piani di Artavaggio population (64.5%). With regard to seed quality, the mean SSM ranged from 39.9 μg (population of Pizzo Arera) to 55.3 μg (Piani di Artavaggio). TSMF ranged from 6.1 mg (Sasso Malascarpa and Piani di Artavaggio) to 11.8 mg (Monte Venturosa). The mean NSF ranged from 110 to 265 (Piani di Artavaggio and Pizzo della Presolana, respectively, Table 1). The proportion of viable seeds was lowest for the populations of Sasso Malascarpa and Corni di Canzo (14.6%) and highest for Grigna Meridionale (48.9%). The mean proportion of seeds that germinated *ex situ* was higher than 80% in all populations, with the exception of Sasso Malascarpa (69.1±7.28%). Seed germination started within 10 d after sowing, and the mean germination percentage plateaued within a month (Fig. S2). The highest mean germination percentages were evident for the populations of Monte Cavallo and Piani di Artavaggio (angular coefficient at the inflection point = 13.1 and 11, respectively), while Sasso Malascarpa exhibited the lowest mean germination percentage (angular coefficient = 4.8).

*Multivariate analyses*

In the PCA, the first two principal axes of variability explained 71.2% of the variance in the data, with elevation being one of the variables contributing strongly to PC1 (PC1 loading = -0.91) (Fig. 2; Table 2). As with elevation, several traits related to pollen and seed quality (PV, PG, SG, SV) and F_IS_ exhibited a strong, negative contribution to variation along PC1 (PC1 loading ≤ -0.70). For PC2, the traits PD, S and FRR all exhibited loadings more negative than -0.70, and R exhibited a positive loading > 0.70. Finally, SSM showed a positive trend with PC1 (PC1 loading = 0.49) and thus the opposite behaviour with respect to SV and SG.

The correlogram of mean values [**Supplementary information** Fig. S3] confirmed pairwise strong positive correlations between PV and elevation and also F_IS_ and elevation (correlation coefficients = 0.94 and 0.81, respectively, p always <0.01) and directly between PV and F_IS_ (correlation coefficient = 0.90, p ≤ 0.05). Other variables with coefficients greater than 0.50 but non-statistically significant were SG, SV, PG and TSMF (positive) and C and FRR (negative). The variables R and S were strongly negatively correlated (correlation coefficient = -0.99, p <0.001).

*Analysis of single traits*

The regression of flowers and rosettes per genet against elevation showed a significant negative relationship (slope = -0.13; p < 0.01). The ANOVA performed on R scores revealed a significant variation between Grigna Meridionale and the other populations (p <0.001; **Supplementary Information** Fig. S1), although this did not follow an elevational gradient, as previously revealed also by the multivariate analyses, with Sasso Malascarpa, Corni di Canzo and Monte Cavallo showing intermediate characteristics between Piani di Artavaggio and Pizzo della Presolana. Regarding pollen quality (viability and germination), both logistic regressions showed significant, positive relationships with elevation (slope = 0.65 and 0.28, respectively, p consistently < 0.001).

The ANOVA performed on single seed mass (SSM) [**Supplementary information** Fig. S4A] and Tukey’s multiple comparison procedure showed a highly significant difference among populations (p < 0.001). The regression of SSM against elevation showed a significant, negative relationship (slope = -2.38, p < 0.001, R^2^_adj_ = 0.18; Fig. S4B), while both TSMF and NSF showed significant but extremely variable increases with elevation, (slope = 1.38 and 0.29, p always < 0.05 and R^2^_adj_ = 0.05 and 0.08, respectively; Fig. 3, Fig. S4C). Finally, both SV and SG showed significant increases with elevation (slope = 0.32 and 0.24, respectively, p always < 0.01).

***Visitor and pollinator assessment***

Of the 123 arthropod taxa distinguished by morphotype, 122 were successfully identified through the molecular approach (1 to the order level, 16 to the family level, 50 to genus level, 55 to species level [**Supplementary information** Table S1]). Most of the collected species were Diptera (68.9%), and in particular Muscidae (26.2%), followed by Hymenoptera (10.7%), represented mainly by Halictidae (3.3%) and Colletidae (2.5%). In particular, among Hymenoptera, *Hoplitis mitis* Nylander (Megachilidae) was found at Corni di Canzo. *Ochlodes sylvanus* Esper was the only representative of Lepidoptera collected by sticky traps. Among Coleoptera, *Cleopomiarus graminis* Gyllenhal (Curculionidae), *Dasytes* sp. (Meliridae), *Meligethes subrugosus* Gyllenhal (Nitidulidae) and *Drilus flavescens* Olivier (Elateridae) were sampled at Piani di Artavaggio and Corni di Canzo, respectively. Finally, *Thrips* spp. (Thysanoptera) were found at almost all sampling sites; mites of two different orders (Trombidiformes, in particular genus *Balaustium*, and Sarcoptiformes) and springtails (Bourletiellidae) were also detected.

The difficulty of distinguishing diagnostic characters from images often hindered the morphological identification of flower visitors to species level. However, observations did confirm the presence of *Apis mellifera*, *Andrena* sp., *Xylocopa* sp., *Lasioglossum* sp., different species of *Bombus* (including *B. hortorum*) and *Hoplitis* sp. (Hymenoptera), *Eupeodes* sp. and *Eristalis* *tenax* L. (Syrphidae, Diptera) and *Coenonympha* sp., *Satyrium* sp. and *Erebia* sp. (Lepidoptera) as pollinators of *C. raineri* flowers [**Supplementary information** Table S1, Fig. S5A-I].

ANOSIM did not reveal significant differences across the insect communities detected at different altitudes, although a slight tendency was evident when considering the entire dataset (degree of dissimilarity R = 0.53, p = 0.07) and pollinators *sensu lato* (R = 0.50; p = 0.13). However, an increase in R statistics and a decrease in p was observed when considering only pollinators *sensu stricto* (R = 0.56; p = 0.07), especially when compared with the results from the non-pollinators dataset (R = 0.14; p = 0.27). Without distinguishing ecological roles, Mantel tests revealed an overall significant difference in the percentage of Diptera (r statistic = 0.71, p = 0.04), Hymenoptera (r statistic = 0.47, p = 0.02), and Lepidoptera (r statistic = 0.49, p = 0.02) with elevation. Linear regression performed at the order level (Fig. 4A, Table 3) showed that, while relative abundance of Acari (Trombidiformes and Sarcoptiformes), springtails (Symphypleona), Coleoptera and Hemiptera tended to remain constant with increasing elevation, Diptera significantly increased (p = 0.01; R^2^_Adj_ = 0.78), and Hymenoptera and Lepidoptera tended to decrease (p = 0.05 and 0.06; R^2^_Adj_ = 0.57 and 0.55, respectively). The predominance of Diptera at high elevation sites (Pizzo della Presolana and Monte Cavallo), as well as the progressive decrease of Hymenoptera and Lepidoptera with increasing elevation was evident also in the within-order taxonomic composition (family level) at sampling sites (Fig. 4B). Regressions of the relative abundance of families against elevation, for hymenopteran and dipteran orders [**Supplementary information** Fig. S6A-D, regression statistics reported in **Supplementary information** Table S2] showed that while Apidae tended to dominate at all elevations over other Hymenoptera [**Supplementary information** Fig. S6A], among Diptera [**Supplementary information** Fig. S6C] Syrphidae and Sarchophagidae tended to decrease and Anthomyiidae and Muscidae tended to increase with increasing elevation (p = 0.01, 0.03, 0.03 and 0.03, respectively [**Supplementary information** Table S2]).

**Discussion**

As with many observations of natural phenomena occurring *in situ*, the analyses demonstrated extensive variability, but did indicate significant associations between elevation and the reproductive ecology of *C. raineri*, with regard to both the phenotypic traits of the species and the taxonomic composition of the pollinator community, supporting our hypotheses. With regard to the inherent traits of *C. raineri*, the allocation of resources to sexual *vs*. vegetative reproduction, as well as pollen and seed quality, changed along the elevational gradient (Hypothesis 1). Indeed, while the general ecological strategy (Grime’s CSR scores) essentially did not vary in relation to elevation, the decreased investment in flowers relative to rosettes with elevation for this rhizomatous perennial suggests that clonal reproduction may be adaptive for this chasmophyte in response to colder and more seasonal conditions (see also Weppler *et al.*, 2006; Arroyo *et al.*, 2017; Körner, 2021).

On the other hand, higher flower production at lower-elevation sites could be a response to sub-optimal conditions (stress-induced flowering, *e.g.* Takeno, 2016). Indeed, despite relatively lower flower production at higher elevations, the quality (in terms of measured viability and germination) of pollen and seeds was higher. Seed germination in particular was considerably lower for the population at the lowest elevation. The trends shown by seed mass and seed germination along the elevation gradient are probably the result of several factors, such as the physiology of the mother plant and the status of the entire population (*e.g.* population size). While seed viability and the initial phase of germination strictly depend on the health of the embryo, seedling development is supported by the endosperm accumulated during the seed filling phase (directly correlated with seed mass; Baskin and Baskin 1998; Martyn *et al.*, 2009). The higher single seed mass measured at lower-elevation sites is thus probably related to the longer duration of the growing season, allowing mother plants to store more reserves in seeds. Here, the mean seed mass was negatively correlated with seed quality (SV and SG), but populations with lower seed mass (Pizzo Arera and Pizzo della Presolana) showed more rapid initiation of germination (at six days, Fig. S2) and at Piani di Artavaggio showed both heavier seeds and delayed germination (on the eleventh day). Therefore, as far as the initial stages of germination are concerned, seed mass is negatively associated with seedling emergence time. This could be due to biophysical constraints and water absorption capacity, as hypothesized for other plant species (Norden *et al.*, 2009). Moreover, as seed production (represented by TSMF and NSF) was positively correlated with elevation and seed mass (SSM) negatively (although extremely variable), at high elevation the fruits contain lighter but more numerous seeds, in agreement with a seed size/number trade-off typical of inter-specific comparisons (*e.g.* Pierce *et al.*, 2014).

For *C. raineri,* pollen viability and germination also increased with elevation, so the higher numbers of seed produced may be facilitated by increased rates of ovule fertilisation. Note that it is difficult to identify all factors affecting fitness in a process as complex as reproductive fitness because seed viability and germination are not the only limiting processes; the subsequent capacity of the seed to support seedling growth, establishment and thus the process of seedling recruitment could also significantly affect population demography, but were beyond the scope of the present study.

Finally, reproductive fitness was not correlated with population density as could be expected (*e.g.*  Hauser *et al.*, 1994; Raijmann *et al.*, 1994; Pierce *et al.,* 2018). Moreover, despite the statistically significant variation of F_IS_ along the elevation gradient, values of inbreeding were extremely low in absolute terms; too low to support a conclusion of an effect on population fitness. Nonetheless, our results suggest that under current climatic conditions *C. raineri* experiences optimal conditions for reproductive fitness at higher-elevation sites (>1,500 m a.s.l.), and is currently limited below this elevation. The low elevation population of Sasso Malascarpa is the smallest (about 30 individuals) and is confined to the most restricted area. This is probably a declining population composed of old individuals that probably have more resources at their disposal due to the longer growing season (as suggested by relatively higher flower production and SSM; Dolezal *et al.*, 2020), but which struggle to produce healthy offspring (indicated by the lower pollen and seed quality), suggesting a process of extinction debt (*e.g.*  Pierce *et al.*, 2018). The population of Corni di Canzo grows at a slightly higher elevation, but its larger size is probably sufficient to maintain high levels of seed germination. However, as it occurs at only a slightly higher elevation, it is possible that the population of Corni di Canzo may face similar problems in coming decades (the short timescale is suggested by the recent extinction at 922 m a.s.l.).

These two populations are genetically closely related both to each other and to the population of Grigna Meridionale (Villa, 2023), which however showed higher seed and pollen quality. Therefore, the reduced reproductive fitness at the lowest elevations (particularly the decrease in seed germination at Sasso Malascarpa) seems not to be due to genetic differences, but could be ascribed to reduced climatic suitability, and, in the long term, reduced population size. Moreover, populations of Sasso Malascarpa and Corni di Canzo already grow at the peak of these reliefs and thus cannot migrate upwards in response to predicted climate change; another reason for concern.

These are issues that also regard mountain chasmophytes in general, which the present study suggests could be expected to undergo immediate limitations to inherent reproductive capacity in the face of climate change at the lowest elevation edge of population ranges. Although the present study represents an instantaneous ‘snapshot’ observation and does not account for factors such as inter-annual variability or direct measurement or modelling of climatic changes (which will form the basis of a separate study), the elevation trends (statistically significant but extremely variable) are evident and the recent recorded case of local extinction in an actively managed protected area at lower altitude is a clear indicator that impacts on reproductive capacity are currently occurring over the relatively short-term scale of years and decades.

Aside from the inherent capacities of the plants themselves, and with regard to the wider reproductive ecology of *C. raineri*, hypothesis 2 (Hymenoptera are the main pollinators of *C. raineri*) was only partially supported: the assessment of insect communities in the vicinity of *C. raineri* individuals at the different sampling sites confirmed that the species is visited mainly by bumblebees, solitary bees (*Xylocopa* sp., *Lasioglossum* sp., *Andrena* sp.; Hymenoptera) and hoverflies (Diptera), especially evident from field monitoring. The detection of *Hoplitis mitis* at Corni di Canzo suggests that this oligolectic species (Brandt *et al.*, 2017; Milet-Pinheiro *et al.,* 2021) may play an important role in the pollination of *C. raineri*, at least at low elevation sites. However, the presence of many generalist taxa (*i.e.* *Bombus* spp.*, Apis mellifera, Andrena* sp.*, Lasioglossum* sp., *Sphecodes geoffrellus* and *Xylocopa* sp. among Hymenoptera and all the detected Diptera and Lepidoptera; Larsson, 2005; see also Dellicour *et al.*, 2015; Lucas *et al.*, 2018) does not allow definition of Rainer’s bellflower as a ''pollination specialist” (*sensu* hypothesis 2).

The insect community, and consequently the pollinator guilds, were shown to change significantly along the elevation gradient, at least at the order level, with a progressive increase of Diptera and a decrease of Hymenoptera and Lepidoptera with altitude, confirming Hypothesis 3 and in general agreement with pollinator shifts evident in other situations (Lefebvre *et al.*, 2018; McCabe and Cobb, 2021, and references therein). In particular, the solitary bees of the genera *Xylocopa*, *Lasioglossum* and *Andrena* were not found at higher elevation sites (Pizzo della Presolana and Monte Cavallo), unlike bumblebees and *Apis mellifera,* supporting the contention that Apidae is the most common hymenopteran family at all elevations (in agreement with Lefebvre *et al.*, 2018). The expected increase of the families Andrenidae and Halictididae (Lefebvre *et al.*, 2018) with elevation was not observed, probably due to the low detection of these taxa. The detection of *Hylaeus nivalis* Morawitz on Pizzo della Presolana is a single but crucial observation, and suggests that this species, specifically sharing the habitat with *C. raineri* (intermediate and high-altitude rocky faces and screes in the Alps; Bossert, 2014) can contribute significantly to the pollination of the species in those contexts where other pollinators may be adversely affected by the low density of flowering plants. In particular, the congeneric species *C.* *barbata* L. is reported to be one of the plant species pollinated by *H. nivalis* (Bossert, 2014). Unfortunately, too little is known about the ecology of this hymenopteran to allow classification as a specialist or generalist pollinator, and the detection only at a single site does not allow verification of the effect of elevation on its distribution. With regard to Diptera, the expected taxonomic shift in favour of Anthomyiidae and Muscidae over Syphidae with increasing altitude (see also Lefebvre *et al.*, 2018; Raguso, 2020) was evident, in addition to a decrease of Sarcophagidae.

Finally, while pollinators differed significantly along the elevational gradient, non-pollinating and opportunistic taxa (Acari, springtails, thrips and beetles) remained constant, meaning that levels of herbivory due to these arthropods are likely to be similar across populations. In particular, the expected decrease of Coleoptera with increasing elevation (Lefebvre *et al.*, 2018) was not evident in our data. This was probably due both to the reduced altitudinal range taken into account, compared to that investigated in the cited literature, and to a possible scarcity of beetle taxa associated with species of the genus *Campanula*. *Cleopomiarus graminis* (Curculionidae) deserves a special mention: this is an oliphagous beetle found at Piani di Artavaggio, whose host plant species belong exclusively to the genera *Campanula*, *Jasione* and *Adenopora* (Campanulaceae; Delbol, 2013; Caldara and Legalov, 2016; Skuhrovec *et al.*, 2018). Although a relationship with elevation cannot be tested with a sporadic observation, the presence of this species is relevant in the wider perspective of the conservation of *C. raineri*. As a specialised pollen-feeding weevil, it could have a major negative impact on the availability of *C. raineri* pollen, affecting reproductive fitness.

**Conclusions**

The climate response of *C. raineri* is strongly mediated by reproductive development. The presence of efficient pollinators such as bumblebees, solitary bees and hoverflies visiting *C. raineri* flowers evidently ensures pollen exchange within populations, as indicated by successful seed production and germination that cannot be ascribed to self-pollination. The lack of evident plant-pollinator specialisation and the presence of occasional visitors and pollen carriers at all elevations, despite belonging to different taxa, ensures pollination at all sites. However, higher-elevation populations of *C. raineri* showed higher reproductive fitness, in terms of both vegetative development (*i.e.* ramet production) and sexual reproduction (pollen and seed viability and germination), suggesting an elevational gradient of environmental suitability for the species. The lowest currently surviving population (<1,200 m a.s.l.) showed evidence of being relictual (*i.e.* formed by fewer individuals with less chance of seedling recruitment) with no possibility of upward migration, and is thus more prone to local extinction due to future climate warming. Conservation actions for *C. raineri* and other rare chasmophytes in the context of climate change should therefore focus on the real possibility of local extinctions in the immediate future and urgently consider *ex-situ* propagation and assisted migration to areas with suitable climatic and habitat conditions.

**Funding**

This work was supported by the Department of Agricultural and Environmental Sciences (DiSAA), University of Milan, Italy [grant number PSR2019_DIP_014SPIER] (Piano di Sostegno alla Ricerca 2019-2021) awarded to S.P. Moreover, S.V. was supported by a Ph.D. Scholarship from the Department of Agricultural and Environmental Sciences, University of Milan, as part of the 35^th^ round of funding in the Doctoral School of Agriculture, Environment and Bioenergy (Dottorato di Ricerca in Agricoltura, Ambiente e Bioenergia).

**Supplementary information**

**Fig. S1** CSR scores of target populations and ANOVA results.

**Fig. S2** Seed germination curves in target populations.

**Fig. S3** Correlogram with Pearson’s correlation coefficients for variables included in the PCA.

**Fig. S4** ANOVA of seed mass, regressions of single seed mass and number of seed per fruit against elevation.

**Fig. S5** Photographic evidence of active pollinators of *C. raineri.*

**Fig. S6** Regressions of pollinating and non pollinating hymenopteran and dipteran families against elevation.

**Table S1** List of arthropods detected at each sampling site.

**Table S2** Regression statistics relating to Fig. S6.

**Acknowledgments:**

The authors would like to thank two anonymous reviewers for their constructive and accurate comments on the original manuscript, which significantly improved the quality of the work. We sincerely thank Dr. Maurizio Mei (Dipartimento di Biologia e Biotecnologie "Charles Darwin" – Entomologia, Università di Roma "Sapienza", Rome, Italy), Enrico Schifani and Daniele Somaggio for helping with the identification of Formicidae and Syrphidae from photographs. We also thank Dr. Cristiano Vernesi (Forest Ecology Unit Research and Innovation Centre, Fondazione Edmund Mach, San Michele all'Adige, Italy) for contribution in developing the initial idea for this study and reading the manuscript, Prof. Bruno E. L. Cerabolini for the helpful suggestions on the populations, strategies and germination of *C. raineri*, Dr. Vittoria Francesca Brambilla (Department of Agricultural and Environmental Sciences, University of Milan, Milan, Italy) for providing PCR reagents and Regione Lombardia for issuing the permit for the collection of *C. raineri* seeds and pollen. As confirmed by Regione Lombardia, no permit was required for arthropods collection pursuant to regional law 10/2008, as the sampling did not involve species included in the annexes of Habitat Directive 92/43/EEC, as well as in the list of species of regional interest as per D.g.r. 7736/2008. Moreover, no special ethical permission was required for target taxa, according to Directive 2010/63/EU.

**Conflict of Interest**

The authors have no conflict of interest to declare.

**Author contributions:**

All the authors conceived and designed the research; S.V. performed all the fieldwork, the main part of laboratory activities and part of data analysis; G.M. supported laboratory activities related to pollinators assessment and performed part of data analysis; S.V. wrote the first draft of the paper and all the authors provided substantial feedback and revisions.

**Data availability statement:**

A preliminary version of this manuscript is part of S.V.’s PhD thesis, available since 3^rd^ Apr. 2023 in the institutional research archive of the University of Milan (AIR Unimi) at the following link: https://air.unimi.it/handle/2434/962757. Data will be made available on the Dryad Digital Repository (https://datadryad.org/stash) on manuscript acceptance. Sequences obtained from barcoding analysis of collected arthropods were submitted to the BOLD system (https://www.boldsystems.org/; sequences ID are reported in **Supplementary information** Table S1).

**Literature cited**:

Aeschimann D, Lauber K, Moser DM, Theurillat J-P. 2004. *Flora alpina*. Bologna: Zanichelli.

Allee WC, Emerson AE, Park O, Park T, Schmidt KP. 1949. *Principles of Animal Ecology*, Philadelphia: W.B. Saunders.

Altschul SF, Gish W, Miller W, Myers EW, Lipman DJ. 1990. Basic local alignment search tool. *Journal of Molecular Biology* 215(3): 403–410. DOI: 10.1016/S0022-2836(05)80360-2.

Arietti N, Fenaroli L. 1963. Campanula raineri *Perpenti endemismo dell’Insubria orientale. Storia reperti e areale*. Bergamo: Ed. Insubriche. [in the Italian language].

Arroyo MTK, Muñoz MS, Henríquez C, Till-Bottraud I, Pérez F. 2006. Erratic pollination, high selfing levels and their correlates and consequences in an altitudinally widespread above-tree-line species in the high Andes of Chile. *Acta Oecologica* 30(2): 248-257. DOI: 10.1016/j.actao.2006.05.006.

Arroyo MTK, Pacheco DA, Dudley LS. 2017. Functional role of long-lived flowers in preventing pollen limitation in a high elevation outcrossing species. *AoB Plants* 9(6): plx050. DOI: 10.1093/aobpla/plx050.

Association of Official Seed Analysis (AOSA), Society of Commercial Seed Technologies (SCST). 2010. Tetrazolium Testing Handbook. AOSA/SCST

Balfour NJ, Castellanos MC, Goulson D, Philippides A, Johnson C. 2022. DoPI: The Database of Pollinator Interactions. *Ecology*: e3801. DOI: 10.1002/ecy.3801.

Baskin CC, Baskin JM. 1998. *Seeds. Ecology, Biogeography, and Evolution of Dormancy and Germination*. New York: Academic Press.

Benson DA, Cavanaugh M, Clark K *et al.* 2013. GenBank. *Nucleic Acids Research* 41(Database issue): D36-42. DOI: 10.1093/nar/gks1195.

Bergmann P, Molau U, Holmgren B. 1996. Micrometeorological impacts on insect activity and plant reproductive success in an alpine environment, Swedish Lapland. *Arctic and Alpine Research* 28(2): 196–202. DOI: 10.2307/1551760.

Billings WD, Mooney HA. 1968. The ecology of arctic and alpine plants. *Biological Reviewers* 43(4): 481-529. DOI: 10.1111/j.1469-185X.1968.tb00968.x.

Bingham RA, Orthner AR. 1998. Efficient pollination of alpine plants. *Nature* 391(6664): 238-239. DOI: 10.1038/34564.

Bjerge K, Mann HMR, Høye TT. 2022. Real-time insect tracking and monitoring with computer vision and deep learning. *Remote Sensing in Ecology and Conservation* 8(3): 315–327. DOI: 10.1002/rse2.245.

Blionis GJ, Vokou D. 2001. Pollination ecology of *Campanula* species on Mt Olympos, Greece. *Ecography* 24(3): 287-297. https://www.jstor.org/stable/3683706.

Bliss LC. 1971. Arctic and Alpine Plant Life Cycles. *Annual Review of Ecology, Evolution, and Systematics* 2: 405-438. https://www.jstor.org/stable/2096935.

Boehme P, Amendt J, Zehner R. 2012. The use of COI barcodes for molecular identification of forensically important fly species in Germany. *Parasitology Research* 110: 2325–2332. DOI: 10.1007/s00436-011-2767-8.

Borcard D, Gillet F, Legendre P. 2011. *Numerical Ecology with R*. New York, NY: Springer New York. DOI: 10.1007/978-1-4419-7976-6.

Borchardt KE, Morales CL, Aizen MA, Toth AL. 2021. Plant–pollinator conservation from the perspective of systems-ecology. *Current Opinion in Insect Science* 47: 154-161. DOI: 10.1016/j.cois.2021.07.003.

Bossert S. 2014. Shelter usage by males of *Hylaeus nivalis* (Morawitz 1867) (Hymenoptera: Apidae) and notes on flower records. *Linzer biologische Beiträge* 46(1): 659-663.

Brandt K, Dötterl S, Francke W, Ayasse M, Milet-Pinheiro P. 2017. Flower visitors of *Campanula*: are oligoleges more sensitive to host-specific floral scents than polyleges? *Journal of Chemical Ecology* 43(1): 4-12. DOI: 10.1007/s10886-016-0802-z.

Brusa G. 2005. Reintroduzione di *Campanula raineri* Perpenti sul Monte Barro (Lecco). In: Rinaldi G, Rossi G, eds. *Orti botanici, conservazione e reintroduzione della flora spontanea in Lombardia*. Regione Lombardia: Quaderni della Biodiversità 2. Centro Regionale per la Flora Autoctona, Scuola Regionale di Ingegneria Naturalistica. [in the Italian language].

Caldara R, Legalov AA. 2016. Systematics of the Holarctic species of the weevil genus *Cleopomiarus* Pierce (Coleoptera: Curculionidae). *Journal of Insect Biodiversity* 4(6): 1-47. DOI: 10.12976/jib/2016.4.6.

Cane J, Minckley RL, Kervin LJ. 2000. Sampling bees (Hymenoptera: Apiformes) for pollinator community studies: Pitfalls of pan-trapping. *Journal of the Kansas Entomological Society* 73(4): 225-231.

Cardoso P, Rigal R, Carvalho J. 2015. BAT - Biodiversity Assessment Tools, an R package for the measurement and estimation of alpha and beta taxon, phylogenetic and functional diversity. *Methods in Ecology and Evolution* 6: 232–236. DOI: 10.1111/2041-210X.12310.

Casazza G, Carta A, Giordani P, Guerrina M, Peruzzi L, Minuto L. 2018. Reproductive biology of the threatened *Lilium pomponium* (Liliaceae), a species endemic to Maritime and Ligurian Alps. *Journal of Plant Research* 131: 633–640. DOI: 10.1007/s10265-018-1019-8.

Chesshire PR, McCabe, LM, Cobb NS. 2021. Variation in plant–pollinator network structure along the elevational gradient of the San Francisco peaks, Arizona. *Insects* 12: 1060. DOI: 10.3390/insects12121060.

Clarke KR, Green RH. 1988. Statistical design and analysis for a 'biological effects' study. *Marine Ecology Progress Series* 46(1-3): 213–226. DOI: 10.3354/meps046213

Cresswell JE, Robertson AW. 1994. Discrimination by pollen-collecting bumblebees among differentially rewarding flowers of an alpine wildflower, *Campanula rotundifolia* (Campanulaceae). *Oikos* 69(2): 304-308. DOI: 10.2307/3546151.

Crowl AA, Miles NW, Visger CJ *et al.* 2016. A global perspective on Campanulaceae: Biogeographic, genomic, and floral evolution. *American Journal of Botany* 103(2): 233-245. DOI: 10.3732/ajb.1500450.

Cui H, Töpper JP, Yang Y, Vandvik V, Wang G 2018. Plastic population effects and conservative leaf traits in a reciprocal transplant experiment simulating climate warming in the Himalayas. *Frontiers in Plant Science* 9: 1069. DOI: 10.3389/fpls.2018.01069.

D’Antraccoli M, Roma‑Marzio F, Benelli G, Canale A, Peruzzi L. 2019. Dynamics of secondary pollen presentation in *Campanula medium* (Campanulaceae). *J Plant Res.* 132(2): 251-261. DOI: 10.1007/s10265-019-01090-1.

Dawson-Glass E, Hargreaves AL. 2022. Does pollen limitation limit plant ranges? Evidence and implications. *Philosophical Transactions of the Royal Society B* 377: 20210014. DOI: 10.1098/rstb.2021.0014.

de Mendiburu F, Yaseen M. 2020. agricolae: statistical procedures for agricultural research. R package version 1.4.0. https://myaseen208.github.io/agricolae/https://cran.r-project.org/package=agricolae.

Delbol M. 2013. Catalogue des Curculionoidea de Belgique (Coleoptera: Polyphaga). *Belgian Journal of Entomology* 13: 1-95. [in the French language].

Dellicour S, Michez D, Mardulyn P. 2015. Comparative phylogeography of five bumblebees: impact of range fragmentation, range size and diet specialization. *Biological Journal of the Linnean Society* 116(4): 926–939. DOI: 10.1111/bij.12636.

Díaz S, Kattge J, Cornelissen J *et al.* 2016. The global spectrum of plant form and function. *Nature* 529: 167–171.

Dolezal J, Kurnotova M, Stastna P, Klimesova J. 2020. Alpine plant growth and reproduction dynamics in a warmer world. *New Phytologist* 228(4): 1295-1305. DOI: 10.1111/nph.16790.

Edgar RC. 2004. MUSCLE: multiple sequence alignment with high accuracy and high throughput. *Nucleic Acids Research* 32(5): 1792-1797. DOI: 10.1093/nar/gkh340.

Eisto A-K, Kuitunen M, Lammi A *et al.* 2000. Population persistence and offspring fitness in the rare bellflower *Campanula cervicaria* in relation to population size and habitat quality. *Conservation Biology* 14(5): 1413-1421. https://www.jstor.org/stable/2641794

Elbrecht V, Leese F. 2015. Can DNA-based ecosystem assessments quantify species abundance? Testing primer bias and biomass-sequence relationships with an innovative metabarcoding protocol. *PLoS ONE* 10(7): e0130324. DOI: 10.1371/journal.pone.0130324.

Eliyahu D, McCall AC, Lauck M, Trakhtenbrot A, Bronstein JL. 2015. Minute pollinators: The role of thrips (Thysanoptera) as pollinators of pointleaf manzanita, *Arctostaphylos pungens* (Ericaceae). *Journal of Pollination Ecology* 16: 64-71.

Erbar C, Leins P. 1995. Portioned pollen release and the syndromes of secondary pollen presentation in the Campanulales-Asterales-complex. *Flora* 190(4): 323-338. DOI: 10.1016/S0367-2530(17)30673-4.

Evette A, Bédécarrats A, Bornette G. 2009. Environmental constraints influence clonal traits of herbaceous plant communities in an alpine massif. *Folia Geobotanica* 44(2): 95-108. https://www.jstor.org/stable/23064497.

Fernández-Pascual E, Carta A, Mondoni A *et al.* 2021. The seed germination spectrum of alpine plants: a global metaanalysis. *New Phytologist* 229(6): 3573–3586. DOI: 10.1111/nph.17086

Flores-Rentería L, Whipple AV, Benally GJ, Patterson A, Canyon B, Gehring CA. 2018. Higher temperature at lower elevation sites fails to promote acclimation or adaptation to heat stress during pollen germination. *Frontiers in Plant Sciences* 9: 536. DOI: 10.3389/fpls.2018.00536.

Frankham R. 1996. Relationship of genetic variation to population size in wildlife. *Conservation* *Biology* 10(6): 1500-1508. https://www.jstor.org/stable/2387021.

García‐Fayos P, Goldarazena A. 2008. The role of thrips in pollination of *Arctostaphyllos uva‐ursi*. *International Journal of Plant Sciences* 169(6): 776-781. https://www.jstor.org/stable/10.1086/588068

Galimberti A, De Mattia F, Bruni I *et al.* 2014. A DNA barcoding approach to characterize pollen collected by honeybees. *PLoS ONE* 9(10): e109363. DOI: 10.1371/journal.pone.0109363.

Geppert C, Bertolli A, Prosser F, Marini L. 2023. Red-listed plants are contracting their elevational range faster than common plants in the European Alps. *Proceedings of the National Academy of Sciences* 120(12): e2211531120. DOI: 0.1073/pnas.2211531120

Grime JP. 1974. Vegetation classification by reference to strategies. *Nature* 250: 26–31. DOI: 10.1038/250026a0

Grime JP. 1977. Evidence for existence of three primary strategies in plants and its relevance to ecological and evolutionary theory. [*The American Naturalist*](https://www.jstor.org/journal/amernatu) 111(982): 1169–1194. https://www.jstor.org/stable/2460262

Grime JP. 2001. Plant strategies, vegetation processes, and ecosystem properties. Chichester: John Wiley & Sons, Second Edition.

Grime JP, Pierce S. 2012. *The evolutionary strategies that shape ecosystems*. Chichester: Wiley-Blackwell.

Harper JL, White J. (1974) The demography of plants. *Annual Review of Ecology, Evolution and Systematics* 5: 419-463 DOI: 10.1146/annurev.es.05.110174.002223

Hartl D.L., Clark AG. 2006. *Principles of Population Genetics*. New York: Oxford University Press.

Hauser TP, Damgaard C, Loeschcke V. 1994. Effects of inbreeding in small plant populations: Expectations and implications for conservation. In: Loeschcke V, Jain SK, Tomiuk J, eds. *Conservation Genetics*. EXS, vol 68. Basel: Birkhäuser. DOI: 10.1007/978-3-0348-8510-2_11.

Hebert PDN, Cywinska A, Ball SL, deWaard JR. 2003. Biological identifications through DNA barcodes. *Proceedings of the Royal Society B: Biological Sciences* 270(1512): 313–321. DOI: 10.1098/rspb.2002.2218.

Hsiao AI, MacGregor ME, Banting JD. 1979. The use of sodium hypochlorite in testing the seed viability of wild oats. *Canadian Journal of Plant Science* 59:1047-1052. DOI: 10.4141/cjps79-164

Inoue K, Maki M, Masuda M. 1996. Evolution of *Campanula* flowers in relation to insect pollinators on islands. In: Lloyd DG, Barrett SCH, eds. *Floral Biology*. Boston: Springer.

Inoue K, Amano M. 2005. Evolution of *Campanula punctata* Lam. in the Izu Islands: changes of pollinators and evolution of breeding systems. *Plant Species Biology* 1(1): 89-97. DOI: 10.1111/j.1442-1984.1986.tb00018.x.

Inouye DW. 2020. Effects of climate change on alpine plants and their pollinators. *Annals of the New York Academy of Sciences Journal.* 1469(1): 26-37. DOI: 10.1111/nyas.14104.

Janzon L-A. 1983. Pollination studies of *Campanula persicifolia* (Campanulaceae) in Sweden. *Grana* 22(3): 153-165. https://doi.org/10.1080/00173138309427701.

Jiang X, Xie Y. 2020. Meta-analysis reveals severe pollen limitation for the flowering plants growing in East Himalaya-Hengduan Mountains region. *BMC Ecology* 20(53). DOI: 10.1186/s12898-020-00322-6.

Johnson LC, Galliart MB, Alsdurf JD, Maricle BR, Baer SG, Bello NM, Gibson DJ, Smith AB. 2022. Reciprocal transplant gardens as gold standard to detect local adaptation in grassland species: New opportunities moving into the 21st century. *Journal of Ecology* 110, 1054–1071. [DOI: 10.1111/1365-2745.13695](https://doi.org/10.1111/1365-2745.13695)

Kahle D, Wickham H. 2013. ggmap: Spatial Visualization with ggplot2. *The R Journal* 5(1): 144-161. http://journal.r-project.org/archive/2013-1/kahle-wickham.pdf

Kearns CA, Inouye DW. 1997. Pollinators, flowering plants, and Conservation Biology. *BioScience* 47(5): 297-307. DOI: 10.2307/1313191.

Khedim N, Poulenard J, Cécillon L, Baudin F, Barré P, Saillard A, Bektaş B, Grigulis K, Lavorel S, Münkemüller T, Choler P. 2023. Soil organic matter changes under experimental pedoclimatic modifications in mountain grasslands of the French Alps. *Geoderma* 429, 116238. DOI: 10.1016/j.geoderma.2022.116238.

Körner C. 2007. The use of ‘altitude’ in ecological research. *Trends in Ecology and Evolution* 22(11): 569-574. DOI: 10.1016/j.tree.2007.09.006.

Körner C. 2021. *Alpine plant life, Functional plant ecology of high mountain ecosystems*. Third Edition. Springer Nature Switzerland AG.

Kozuharova E, Lavchiev V, Lennartsson T. 2005. Flies of the genus *Thricops* (Muscidae, Diptera) as pollinators of plants with bell-shaped flowers in the mountains of Bulgaria. *Studia Dipterologica* 12: 409-418.

Kumar S, Stecher G, Li M, Knyaz C, Tamura K. 2018. MEGA X: Molecular Evolutionary Genetics Analysis across computing platforms. *Molecular Biology and Evolution* 35(6): 1547-1549. DOI: 10.1093/molbev/msy096.

Johnson LC, Galliart MB, Alsdurf JD *et al.* 2022. Reciprocal transplant gardens as gold standard to detect local adaptation in grassland species: New opportunities moving into the 21st century. *Journal of Ecology* 110(5):1054-1071. DOI: 10.1111/1365-2745.13695.

Lara-Romero C, Seguí J, Pérez-Delgado A, Nogales M, Traveset A. 2019. Beta diversity and specialization in plant–pollinator networks along an elevational gradient. *Journal of Biogeography* 46(7): 1598-1610. DOI: 10.1111/jbi.13615.

Larsson M. 2005. Higher pollinator effectiveness by specialist than generalist flower-visitors of unspecialized *Knautia arvensis* (Dipsacaceae). *Oecologia* 146(3): 394-403. https://www.jstor.org/stable/20062514.

Lefebvre V, Villemant C, Fontaine C, Daugeron C. 2018. Altitudinal, temporal and trophic partitioning of flower-visitors in Alpine communities. *Scientific Reports* 8: 4706. DOI: 10.1038/s41598-018-23210-y.

Lucas A, Bodger O, Brosi BJ *et al.* 2018. Generalisation and specialisation in hoverfly (Syrphidae) grassland pollen transport networks revealed by DNA metabarcoding. *Journal of Animal Ecology* 87(4): 1008-1021. DOI: 10.1111/1365-2656.12828.

Maechler M, Rousseeuw P, Croux C *et al.* 2023. robustbase: Basic Robust Statistics. R package version 0.99-1. http://robustbase.r-forge.r-project.org/.

Magoga G, Sahin DC, Fontaneto D, Montagna M. 2018. Barcoding of Chrysomelidae of Euro-Mediterranean area: efficiency and problematic species. *Scientific Reports* 8(1): 13398. DOI: 10.1038/s41598-018-31545-9.

Martyn AJ, Seed LU, Ooi MKJ, Offord CA. 2009. Seed fill, viability and germination of NSW species in the family Rutaceae. *Cunninghamia* 11(2): 203-212.

McCabe L, Cobb NS. 2021. From bees to flies: global shift in pollinator communities along elevation gradients. *Frontiers in Ecology and Evolution* 8: 626124. DOI: 10.3389/fevo.2020.626124

Mereghetti V, Chouaia B, Limonta L, Locatelli DP, Montagna M. 2019. Evidence for a conserved microbiota across the different developmental stages of *Plodia interpunctella*. *Insect Science* 26: 466-478. DOI: 10.1111/1744-7917.12551.

Milet-Pinheiro P, Ayasse M, Dobson HEM, Schlindwein C, Francke W, Dötterl S. 2013. The chemical basis of host-plant recognition in a specialized bee pollinator. *Journal of Chemical Ecology* 39(11-12): 1347–1360. DOI: 10.1007/s10886-013-0363-3.

Milet-Pinheiro P, Ayasse M, Dötterl S. 2015. Visual and olfactory floral cues of *Campanula* (Campanulaceae) and their significance for host recognition by an oligolectic bee pollinator. *PLoS ONE* 10(6): e0128577. DOI: 10.1371/journal.pone.0128577.

Milet-Pinheiro P, Santos PSC, Prieto-Benítez S, Ayasse M, Dötterl S. 2021. Differential evolutionary history in visual and olfactory floral cues of the bee-pollinated genus *Campanula* (Campanulaceae). *Plants* 10: 1356. DOI: 10.3390/plants10071356.

Minachilis K, Kantsa A, Devalez J, Trigas P, Tscheulin T, Petanidou T. 2020. Bumblebee diversity and pollination networks along the elevation gradient of Mount Olympus, Greece. *Diversity and Distribution* 26(11): 1566–1581. DOI: [10.1111/ddi.13138](https://doi.org/10.1111/ddi.13138).

Montagna M, Sassi D, Giorgi A. 2013. *Pachybrachis holerorum* (Coleoptera: Chrysomelidae: Cryptocephalinae), a new species from the Apennines, Italy, identified by integration of morphological and molecular data. *Zootaxa* 3741(2): 243-253. DOI: 10.11646/zootaxa.3741.2.3.

Mound LA, Teulon DAJ. 1995. Thysanoptera as phytophagous opportunists. In: Parker BL, Skinner M, Lewis T, eds. *Thrips biology and management*. NATO ASI Series, 276. Boston: Springer. DOI: 10.1007/978-1-4899-1409-5_1.

Murashige T, Skoog F. 1962. A revised medium for rapid growth and bioassays with tobacco tissue cultures. *Physiologia Plantarum* 15(3): 473-497. DOI: 10.1111/j.1399-3054.1962.tb08052.x

Neal PR, Dafni A, Giurfa M. 1998. Floral symmetry and its role in plant-pollinator systems: Terminology, distribution, and hypotheses. *Annual Review of Ecology and Systematics* 29: 345-373. DOI: 10.1146/annurev.ecolsys.29.1.345.

Nobis M, Klichowska E, Vintsek L, Wróbel A, Nobis A, Zalewska-Gałosz J, Nowak A. 2023. Evolutionary response of cold-adapted chasmophytic plants to Quaternary climatic oscillations in the Mountains of Central Asia (a world hotspot of biodiversity). *Diversity and Distributions* 29: 1458–1477. DOI: 10.1111/ddi.13773

Nyman Y. 1992. Pollination mechanisms in six *Campanula* species (Campanulaceae). *Plant Systematics and Evolution* 181(1-2): 97-108. https://www.jstor.org/stable/ 23674787.

Oksanen J, Simpson GL, Blanchet FG *et al.* 2017. Vegan: Community Ecology Package. R package Version 2.4-3. https://cran.r-project.org/web/packages/vegan/vegan.pdf

Olesen JM, Alarcón M, Ehlers BK, Aldasoro JJ, Roquet C. 2012. Pollination, biogeography and phylogeny of oceanic island bellflowers (Campanulaceae). *Perspectives in Plant Ecology, Evolution and Systematics* 14(3): 169-182. DOI: 10.1016/j.ppees.2012.01.003.

Padgham M, Rudis B, Lovelace R, Salmon M. 2017. Osmdata. *Journal of Open Source Software* 2(14): 305. DOI: 10.21105/joss.00305.

Pierce S, Negreiros D, Cerabolini BEL *et al.* 2017. A global method for calculating plant CSR ecological strategies applied across biomes world-wide. *Functional Ecology* 31(2): 444-457. DOI: 10.1111/1365-2435.12722.

Pierce S, Spada A, Caporali E *et al.* 2018. Identifying population thresholds for flowering plant reproductive success: the marsh gentian (*Gentiana pneumonanthe*) as a flagship species of humid meadows and heathland. *Biodiversity and Conservation* 27: 891–905. DOI: 10.1007/s10531-017-1470-1.

Pignatti S. 2018. *Flora d’Italia*, 3. Bologna: Edagricole.

Pinheiro JC, Bates DM. 2000. *Mixed-Effects Models in S and S-PLUS*. New York: Springer. DOI: 10.1007/b98882.

Pinheiro J, Bates D, R Core Team. 2023. nlme: linear and nonlinear mixed effects models. R package version 3.1-163. https://CRAN.R-project.org/package=nlme.

Podani J, Kalapos T, Barta B, Schmera D. Principal component analysis of incomplete data – A simple solution to an old problem. *Ecological Informatics* 61:101235. DOI: 10.1016/j.ecoinf.2021.101235.

R Core Team. 2022. R: A language and environment for statistical computing. Vienna: R Foundation for Statistical Computing. https://www.R-project.org/.

Raguso RA. 2020. Don’t forget the flies: dipteran diversity and its consequences for floral ecology and evolution. *Applied Entomology and Zoology* 55: 1-7. DOI: 10.1007/s13355-020-00668-9.

Raijmann LEL, van Leeuwen NC, Kersten R, Oostermeijer JGB, den Nijs HCM, Menken SBJ. 1994. Genetic variation and outcrossing rate in relation to population size in *Gentiana pneumonanthe* L. *Conservation Biology* 8: 1014-1026.

Ramos-Jiliberto R, Domínguez D, Espinoza C *et al.* 2010. Topological change of Andean plant–pollinator networks along an altitudinal gradient. *Ecological Complexity* 7(1): 86–90. https://doi.org/10.1016/j.ecocom.2009.06.001.

Ratnasingham S, Hebert PDN. 2013. Bold: The Barcode of life data system (http://www.barcodinglife.org). *Molecular Ecology Notes* 7(3): 355–364. https://doi.org/10.1111%2Fj.1471-8286.2007.01678.x” \t "_blank.

Raunkiær CC. 1934. *The life forms of plants and statistical plant geography*. Oxford: Oxford University Press.

Revelle W. 2022. psych: procedures for psychological, psychometric, and personality research. R package version 2.2.9. https://CRAN.R-project.org/package=psych.

Rinker TW, Kurkiewicz D. 2017. *pacman: Package Management for R*. http://github.com/trinker/pacman

Ritz C, Baty F, Streibig JC, Gerhard D. 2015. Dose-response analysis using R. *PLOS ONE*, 10(12): e0146021. DOI: 10.1371/journal.pone.0146021.

Rumpf S, Hülber K, Klonner G, Moser D, Schütz M, Wessely J, Willner W, Zimmermann N, Dullinger S. 2018. Range dynamics of mountain plants decrease with elevation. *Proceedings of the National Academy of Sciences* 115: 1848-1853.

Schlindwein C, Wittmann D, Martins CF *et al.* 2005. Pollination of *Campanula rapunculus* L. (Campanulaceae): How much pollen flows into pollination and into reproduction of oligolectic pollinators? *Plant Systematics and Evolution* 250: 147–156. DOI: 10.1007/s00606-004-0246-8.

Skuhrovec J, Gosik R, Caldara R, Toše*vs*ki I, Łętowski J, Szwaj E. 2018. Morphological characters of immature stages of Palaearctic species of *Cleopomiarus* and *Miarus* and their systematic value in Mecinini (Coleoptera, Curculionidae, Curculioninae). *ZooKeys* 808: 23–92. DOI: 10.3897/zookeys.808.28172.

Sperotto RA, Grbic V, Pappas ML *et al.* 2019. Editorial: plant responses to phytophagous mites/thrips and search for resistance. *Frontiers in Plant Science Plant Pathogen Interactions* 10: 866. DOI: 10.3389/fpls.2019.00866.

Sulusoglu M, Cavusoglu A. 2014. In vitro pollen viability and pollen germination in cherry laurel (*Prunus laurocerasus* L.). *The Scientific World Journal* 2014: 657123. DOI: 10.1155/2014/657123.

Tito R, Vasconcelos HL, Feeley KJ. 2020. Mountain ecosystems as natural laboratories for climate change experiments. *Frontiers in Forests and Global Change* 3(38). DOI: 10.3389/ffgc.2020.00038.

Totland Ø. 2001. Environment-dependent pollen limitation and selection on floral traits in an alpine species. *Ecology* 82(8): 2233-2244. DOI: [10.1890/0012-9658(2001)082[2233:EDPLAS]2.0.CO;2](https://doi.org/10.1890/0012-9658(2001)082%5b2233:EDPLAS%5d2.0.CO;2).

Vera ML. 1997. Effects of altitude and seed size on germination and seedling survival of heathland plants in North Spain. *Plant Ecology* 133(1): 101-106. https://www.jstor.org/stable/20050545.

Villa S, Ceriani RM, Cerabolini BEL, Pierce S. 2021. Germination response across populations of the stenoendemic chasmophyte *Campanula raineri* (Campanulaceae). *Flora Mediterranea* 31: 267-270. DOI: 10.7320/FlMedit31.267.

Villa S. 2023. Population genomics, distribution and reproductive ecology of an Italian steno-endemic chasmophyte, *Campanula raineri* [PhD Thesis]. University of Milan. https://hdl.handle.net/2434/962757

Vranken S, Brys R, Hoffmann M, Jacquemyn H. 2014. Secondary pollen presentation and the temporal dynamics of stylar hair retraction and style elongation in *Campanula* *trachelium* (Campanulaceae). *Plant Biology* 16(3): 669-676. DOI: 10.1111/plb.12097.

Warren SD, Harper KT, Booth GM. 1988. Elevational distribution of insect pollinators. *The American Midland Naturalist Journal* 120(2): 325-330. DOI: 10.2307/2426004.

Weppler T, Stoll P, Stocklin J. 2006. The relative importance of sexual and clonal reproduction for population growth in the long-lived alpine plant *Geum reptans*. *Journal of Ecology* 94(4): 869-879. https://www.jstor.org/stable/3879553.

Wickham H .2011. The split-apply-combine strategy for data analysis. *Journal of Statistical Software* 40(1):1-29. https://www.jstatsoft.org/v40/i01/.

Wickham H. 2016. *ggplot2: Elegant graphics for data analysis*. New York: Springer-Verlag. https://ggplot2.tidyverse.org.

Yucedag C, Cetin M, Baris Ozel H, Easa Saad Abo Aisha A, Muragaa Alrabiti OB, Aljama AMO. 2021. The impacts of altitude and seed pretreatments on seedling emergence of Syrian juniper (*Juniperus* *drupacea* (Labill.) Ant. et Kotschy). *Ecological Processes* 10(7). DOI: 10.1186/s13717-020-00276-z.

Zu K, Wang Z, Zhu X, Lenoir J, Shrestha N, Lyu T, Luo A, Li Y, Ji C, Peng S, Meng J, Zhou J. 2021. Upward shift and elevational range contractions of subtropical mountain plants in response to climate change. *Science of the Total Environment* 783: 146896. DOI: 10.1016/j.scitotenv.2021.146896.

**List of captions:**

**Fig. 1** A) Map of sampling sites (WGS84 coordinate system) and B) showy flowering of *C. raineri* on calcareous cliffs at Pizzo Arera. Black dots indicate sampling sites: SM = Sasso Malascarpa, CA = Corni di Canzo, GM = Grigna Meridionale, RG = Monte Resegone, AV = Piani di Artavaggio, VE = Monte Venturosa, AR = Pizzo Arera, CV = Monte Cavallo, PS = Pizzo della Presolana. The map was produced in R using the following packages: *ggmap, ggplot2, osmdata, pacman* (Kahle and Wickham, 2013; Wickham, 2016; Padgham *et al.*, 2017; Rinker and Kurkiewicz, 2017). C) Location of the study site. The red box indicates the geographical range of *C. raineri* (map of Italy modified from https://d-maps.com/carte.php?num_car=2327&lang=en). Photograph of *C. raineri* by Simon Pierce.

**Fig. 2** Principal component analysis (PCA) of phenotypic traits, population density (PD), inbreeding coefficient (F_IS_), flowers/reproducing structures ratio (FRR) and mean elevation of populations (grey arrows) measured for *C. raineri* in 9 sampling sites. Phenotypic traits included: SG = seed germination, SV = seed viability, SSM = single seed mass, TSMF = total seed mass per fruit (in green), PG = pollen germination, PV = pollen viability (in red), C = competitivity, S = stress-tolerance, R = ruderality (in blue). Sampling sites (indicated by black dots): SM = Sasso Malascarpa, CA = Corni di Canzo, PS = Pizzo della Presolana, VE = Monte Venturosa, RG = Monte Resegone, AV = Piani di Artavaggio, GM = Grigna Meridionale, CV = Monte Cavallo). *x* and *y* axes represent the first and the second Principal Component, respectively, with the relative proportion of explained variance in brackets. Note that PCA loadings were rescaled by a factor of 0.3 for clearer visualisation. PCA loadings are reported in Table 2.

**Fig. 3** Linear regression of total seed mass per fruit (TSMF) against elevation. The mean elevation of sampling sites was modelled as an independent variable. Dots represent replicates for different sampling sites (SM = Sasso Malascarpa, CA = Corni di Canzo, RG = Monte Resegone, GM = Grigna Meridionale, AV = Piani di Artavaggio, PS = Pizzo della Presolana, VE = Monte Venturosa, AR = Pizzo Arera, CV = Monte Cavallo). Dashed-lines: 95% C.I. Slope: 1.38, p < 0.02; R^2^_Adj_ = 0.05, F = 5.85, regression equation: $y=1.38x+8.87$.

**Fig. 4** Relative abundance of arthropod orders and families over the total number of detected taxa at sampling sites. A). Regressions of the relative abundance of orders (*y* axis) against elevation (*x* axis). Asterisks indicate significant regressions. B). Barplot of the taxonomic composition at the family level (expressed as relative abundance, *x* axis) of detected orders at sample sites. For A). different colours and symbols indicate orders, points indicate the relative abundance of each order as detected at different sampling sites, ranked by increasing mean elevation (sites: SM = Sasso Malascarpa, CA = Corni di Canzo, GM = Grigna Meridionale, AV = Piani di Artavaggio, PS = Pizzo della Presolana, CV = Monte Cavallo). Regression statistics are reported in Table 3; for B). *y* axis: SM = Sasso Malascarpa, CA = Corni di Canzo, GM = Grigna Meridionale, AV = Piani di Artavaggio, PS = Pizzo della Presolana, CV = Monte Cavallo. TRO = Trombidiformes, THY = Thysanoptera, SYM. = Symphypleona, SAR. = Sarcoptiformes, LEP. = Lepidoptera, HYM. = Hymenoptera, HEM. = Hemiptera, DIP. = Diptera, COL. = Coleoptera; taxa determined only to the level of order are indicated with the order name followed by ND (Not Determined). Different colours represent different families.

**Tables**

**Table 1** A) Summary table of mean values for variables measured for target populations of *C. raineri* (in order of increasing elevation) to assess reproductive fitness (FRR = Flowers/reproducing structures ratio, C = competitivity, S = stress-tolerance, R = ruderality, PD = population density, PV = pollen viability, PG = pollen germination, SSM = single seed mass, TSMF = total seed mass per fruit, SV = seed viability, SG = seed germination, F_IS_ = inbreeding coefficient). When available, the standard error (s.e.) of mean values is reported. For all sampling sites, elevation and geographical coordinates (WGS84) are reported, as well as the acronym used in analyses (Code). B) Summary table of totals for each variable.

| **A** | | | | | | | | | | | | | | | | |
| --- | --- | --- | --- | --- | --- | --- | --- | --- | --- | --- | --- | --- | --- | --- | --- | --- |
| **Site** | **Code** | **Latitude (˚N)** | **Longitude**  **(˚E)** | **Elevation (m a.s.l.)** | **FRR** | **C (%)±s.e.** | **S (%)±s.e.** | **R (%)±s.e.** | **PD (ind. m^-2^)** | **PG (%)±s.e.** | **PV (%)±s.e.** | **SSM (µg)±s.e.** | **TSMF (mg)±s.e.** | **SV (%)** | **SG (%)±s.e.** | **FIS (× 10^-4^)** |
| Sasso Malascarpa | SM | 45.8503 | 9.3181 | 1,159 | 0.25±0.06 | 7.7±1.07 | 3.2  ±2.67 | 89.1±2.38 | 0.03 | 36.1±3.64 | 44.7±2.64 | 52.7±1.12 | 6.1±1.08 | 14.58 | 69.1±7.28 | -6.8 |
| Corni di Canzo | CA | 45.8626 | 9.3229 | 1,226 | 0.13±0.03 | 9.1±0.63 | 6.4±2.75 | 84.5±2.37 | 0.45 | 46.4±1.84 | 61.1±2.82 | 48.3±0.99 | 6.8±3.91 | 14.58 | 86.12±1.00 | -6.6 |
| Monte Resegone | RG | 45.8582 | 9.4889 | 1,645 | NA | NA | NA | NA | 0.42 | NA | NA | 46.7±0.60 | 8.0±1.36 | 27.66 | 82.6±1.61 | -5.2 |
| Grigna Meridionale | GM | 45.9133 | 9.3944 | 1,728 | 0.23±0.02 | 5.5±0.48 | 18.7±1.50 | 75.8±1.25 | 0.73 | 44.9±3.35 | 79.1±4.08 | 44.5±0.21 | 8.6±1.41 | 48.89 | 83.3±0.81 | -6 |
| Piani di Artavaggio | AV | 45.9413 | 9.5367 | 1,789 | 0.17±0.06 | 7.0±0.50 | 14.5±1.85 | 78.6±1.51 | 0.17 | 64.5±2.95 | 75.9±4.18 | 55.3±0.44 | 6.1±0.81 | 28.57 | 82.7±2.88 | -5.6 |
| Pizzo della Presolana | PS | 45.9475 | 10.0736 | 1,856 | 0.1±0.02 | 7.6±0.36 | 2.7±1.54 | 89.7±1.48 | 0.01 | 47.7±5.53 | 72.5±2.30 | 42.3±0.71 | 11.2±1.56 | 31.58 | 82.4±1.51 | -5.6 |
| Monte Venturosa | VE | 45.9272 | 9.6168 | 1,885 | NA | NA | NA | NA | 0.23 | NA | NA | 53.4±1.02 | 11.8±2.12 | 17.78 | 80.2±2.03 | -4.9 |
| Pizzo Arera | AR | 45.9292 | 9.8057 | 1,934 | NA | NA | NA | NA | 0.20 | NA | NA | 39.9±0.34 | 7.5±1.39 | 36.36 | 86.1±1.58 | -4.3 |
| Monte Cavallo | CV | 46.0358 | 9.6940 | 2,130 | 0.1±0.02 | 6.5±0.40 | 9.0±2.24 | 84.6±2.03 | 0.13 | 63.7±1.15 | 93.6±1.08 | 44.0±0.11 | 11.0±1.96 | 28.26 | 90.4±1.21 | -5.3 |

| **B** | | | | | | | | | |
| --- | --- | --- | --- | --- | --- | --- | --- | --- | --- |
| **Site** | **Locally estimated population size** | **Number of genets considered for FRR** | **Total number of pollen grains used for PG calculation** | **Total number of pollen grains used for PV calculation** | **Total number of seeds used for SV calculation** | **Total number of seeds used for SG calculation** | **Estimated number of collected seeds** | **Number of collected fruits** | **mean NSF±s.e.** |
| Sasso Malascarpa | 30 | 15 | 3,232 | 5,866 | 48 | 355 | 7,723 | 65 | 116.6±20.47 |
| Corni di Canzo | 250 | 31 | 2,445 | 9,230 | 48 | 454 | 6,324 | 31 | 140.5±81.1 |
| Monte Resegone | 300 | NA | NA | NA | 47 | 443 | 16,399 | 95 | 170.7±29.10 |
| Grigna Meridionale | 300 | 73 | 4,923 | 10,310 | 45 | 174 | 20,550 | 132 | 192.8±31.74 |
| Piani di Artavaggio | 50 | 3 | 4,774 | 14,373 | 49 | 361 | 11,631 | 112 | 109.7±14.72 |
| Pizzo della Presolana | 200 | 69 | 7,092 | 14,988 | 38 | 279 | 24,476 | 101 | 265.4±36.94 |
| Monte Venturosa | 50 | NA | NA | NA | 45 | 548 | 9,678 | 62 | 221.6±39.67 |
| Pizzo Arera | 200 | NA | NA | NA | 55 | 197 | 11,338 | 69 | 187.2±34.81 |
| Monte Cavallo | 50 | 47 | 3,979 | 23,735 | 46 | 412 | 16,848 | 67 | 248.7±44.55 |

**Table 2** PCA loadings for the two main components of the variables included in the analysis (elevation, SG = seed germination, SV = seed viability, PG = pollen germination, PV = pollen viability, PD = population density, C = competitivity, S = stress-tolerance, R = ruderality, FIS = inbreeding coefficient, SSM = single seed mass, TSMF = total seed mass per fruit, FRR = flowers/reproducing structures ratio) calculated according to Podani *et al.* (2021). Loadings with absolute values ≥ 0.70 and ≤ -0.70 are highlighted in bold font. PC1 and PC2 explained 45.4% and 25.8% of the variance, respectively.

|  | **PC1** | **PC2** |
| --- | --- | --- |
| **Elevation** | **-0.91** | 0.32 |
| **SG** | **-0.80** | 0.23 |
| **SV** | **-0.71** | -0.44 |
| **PG** | **-0.72** | 0.26 |
| **PV** | **-0.98** | 0.13 |
| **PD** | -0.25 | **-0.74** |
| **C** | 0.67 | 0.46 |
| **S** | -0.60 | **-0.77** |
| **R** | 0.54 | **0.78** |
| **FIS** | **-0.70** | 0.31 |
| **SSM** | 0.49 | -0.22 |
| **TSMF** | -0.49 | 0.52 |
| **FRR** | 0.46 | **-0.78** |

**Table 3** Regression statistics of the relative abundance of detected arthropod orders with elevation, relating to Fig. 4. P-values ≤ 0.05 are marked with an asterisk.

| **Order** | **slope** | **R^2^_Adj_** | **F** | **p** | **Regression equation** |
| --- | --- | --- | --- | --- | --- |
| Diptera | 0.04 | 0.78 | 19.03 | 0.01* | $y=0.04x-13.8$1 |
| Hymenoptera | -0.02 | 0.57 | 7.57 | 0.05* | $y=-0.02x+61.24$ |
| Lepidoptera | -0.01 | 0.55 | 7.05 | 0.06 | $y=-0.01x+20.03$ |
| Hemiptera | < 0.01 | 0.12 | 1.69 | 0.26 | $y=-0.002x+4.31$ |
| Thysanoptera | -0.01 | 0.54 | 6.93 | 0.06 | $y=-0.01x+25.07$ |
| Sarcoptiformes | < 0.01 | -0.24 | 0.04 | 0.85 | $y=0.0005x-0.08$ |
| Symphypleona | < 0.01 | 0.23 | 2.53 | 0.19 | $y=0.004x-6.26$ |
| Coleoptera | < 0.01 | -0.25 | 0.01 | 0.93 | $y=-0.0007x+7.99$ |
| Trombidiformes | < 0.01 | -0.20 | 0.16 | 0.71 | $y=0.002x+1.46$ |
