# Supplementary material for: Elevational shifts in reproductive ecology indicate the climate response of a model chasmophyte, Rainer’s bellflower (Campanula raineri)
Source: Ann Bot. 2024 Sep 30;135(1-2):181–98. doi: 10.1093/aob/mcae164 (PMC11805931; doi:10.1093/aob/mcae164)
Supplement: mcae164_suppl_Supplementary_Data [file mcae164_suppl_supplementary_data.zip › aob-24018-s05.docx]

Two hoverflies (Syrphidae, Diptera) visiting flowers of *Campanula raineri* Perp. (Campanulaceae), stenoendemic chasmophyte of the Italian Prealps. Together with bees and bumblebees, hoverflies are important generalist pollinators. Photo by Sara Villa, Monte Cavallo (BG, Italy), July 2020.
